# Exploring Structural–Photophysical Property Relationships in Mitochondria-Targeted Deep-Red/NIR-Emitting Coumarins

**DOI:** 10.3390/ijms242417427

**Published:** 2023-12-13

**Authors:** Eduardo Izquierdo-García, Anna Rovira, Joan Forcadell, Manel Bosch, Vicente Marchán

**Affiliations:** 1 Secció de Química Orgànica, Departament de Química Inorgànica i Orgànica, Institut de Biomedicina de la Universitat de Barcelona (IBUB), Universitat de Barcelona (UB), Carrer Martí i Franquès 1-11, E-08028 Barcelona, Spain; 2 Unitat de Microscòpia Òptica Avançada, Centres Científics i Tecnològics de la Universitat de Barcelona (CCiTUB), Universitat de Barcelona (UB), Avinguda Diagonal 643, E-08028 Barcelona, Spain

**Keywords:** coumarin, COUPY, bioimaging, organic fluorophore, mitochondria

## Abstract

Organic fluorophores operating in the optical window of biological tissues, namely in the deep-red and near-infrared (NIR) region of the electromagnetic spectrum, offer several advantages for fluorescence bioimaging applications owing to the appealing features of long-wavelength light, such as deep tissue penetration, lack of toxicity, low scattering, and reduced interference with cellular autofluorescence. Among these, COUPY dyes based on non-conventional coumarin scaffolds display suitable photophysical properties and efficient cellular uptake, with a tendency to accumulate primarily in mitochondria, which renders them suitable probes for bioimaging purposes. In this study, we have explored how the photophysical properties and subcellular localization of COUPY fluorophores can be modulated through the modification of the coumarin backbone. While the introduction of a strong electron-withdrawing group, such as the trifluoromethyl group, at position 4 resulted in an exceptional photostability and a remarkable redshift in the absorption and emission maxima when combined with a julolidine ring replacing the *N*,*N*-dialkylaminobenzene moiety, the incorporation of a cyano group at position 3 dramatically reduced the brightness of the resulting fluorophore. Interestingly, confocal microscopy studies in living HeLa cells revealed that the 1,1,7,7-tetramethyl julolidine-containing derivatives accumulated in the mitochondria with much higher specificity. Overall, our results provide valuable insights for the design and optimization of new COUPY dyes operating in the deep-red/NIR region.

## 1. Introduction

Organic fluorophores with operability in the optical window of biological tissues have gained much attention in recent years for their broad biological applications [1]. The optical window of biological tissues, also known as the near-infrared (NIR) window, refers to the region of the electromagnetic spectrum spanning from approximately 650 to 1350 nm, and it can be subdivided into three main sub-regions: deep-red (640–700 nm), NIR I (700–900 nm), and NIR II (950–1350 nm) [2,3]. Deep-red/NIR light offers many advantages over shorter wavelength radiation, including minimum photodamage to living cells, deep tissue penetration, and a higher signal-to-noise ratio owing to a lower interference with the autofluorescence of biomolecules present in cells [3]. Accordingly, organic fluorophores operating in this region are widely used for diagnostic and therapeutic applications, such as biomedical imaging [4], biosensing [1], fluorescence-guided surgery [5], photodynamic therapy (PDT) [6], photothermal therapy (PTT) [7] and photoactivated chemotherapy (PACT) [8,9].

**Figure 1:**
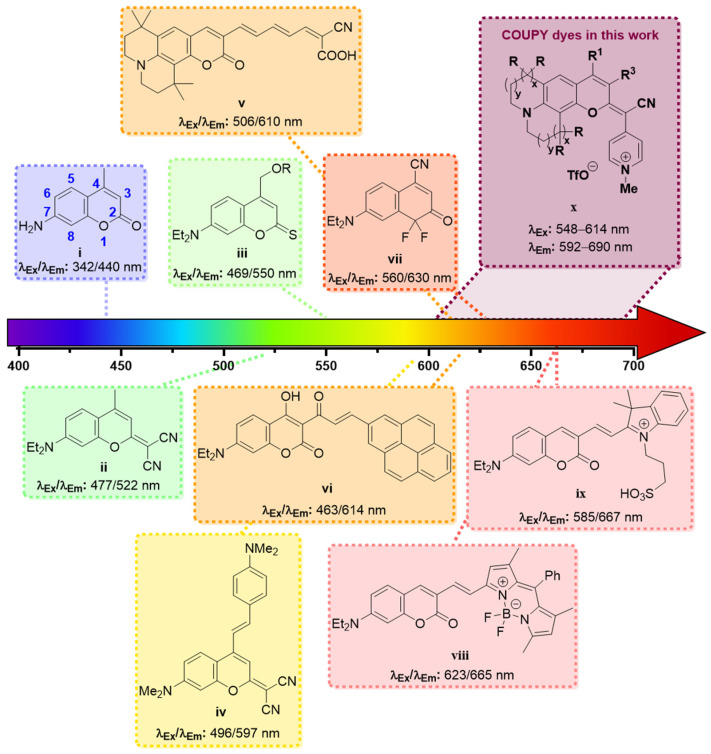
Examples of coumarin-based fluorophores with increasingly redshifted absorption and emission. Refs: coumarin i [13], ii [14], iii [15], iv [16], v [17], vi [18], vii [19], viii [20], ix [21].

Among all the existing organic dyes, coumarins are particularly well suited for bioimaging due to their favorable photophysical properties, excellent cell permeability, and synthetic accessibility [3]. Although the unsubstituted coumarin (i.e., 2*H*-chromen-2-one) absorbs in the UV region and is almost non-fluorescent [10], the photophysical properties of coumarin dyes can easily be tuned by decorating the core scaffold with the appropriate substituents. In this regard, several coumarin-based dyes have been reported over the last decades (Figure 1), and significant efforts have been devoted to the analysis, understanding, and optimization of their photophysical properties [3,11,12].

As a result of these studies, it has been possible to establish some accurate structure–property relationships to help chemists design new coumarin derivatives with improved spectroscopic properties. The rule of thumb in the molecular design of coumarin-based dyes is that the introduction of electron-donating groups (EDG) at positions 6 or 7 (e.g., –OH, –NRR’) and electron-withdrawing groups (EWG) at positions 3 or 4 (e.g., –CF_3_, –COOR, –CN) results in a redshift of the absorption and the emission maxima, due to the existence of a strong intramolecular charge transfer (ICT) process along the coumarin backbone [3]. Extending the *π*-conjugation system via the fusion of polymethine groups [17] or bulky aromatic scaffolds (e.g., pyrene [18,22], hemicyanine [21,23,24,25], or BODIPY [20,26,27]) through position 3 is another common strategy to redshift the absorption and emission band of coumarins. Moreover, several modifications of the lactone moiety have been reported to induce an additional bathochromic effect. For instance, the thionation of the lactone carbonyl oxygen at position 2 lead to an ~80 nm redshift in the absorption and the emission maxima of a 7-(*N*,*N*-dialkylamino) coumarin [15]. A similar effect could be achieved through the incorporation of a dicyanomethylene group at position 2 [14,28]. Remarkably, extending the conjugation of the π-system by introducing a styryl moiety at position 4 resulted in a further bathochromic shift [16]. NIR-emitting coumarin-based fluorophores have also been obtained by replacement of the *O*–atom at position 1 with a difluoromethylene group [19].

**Scheme 1:**
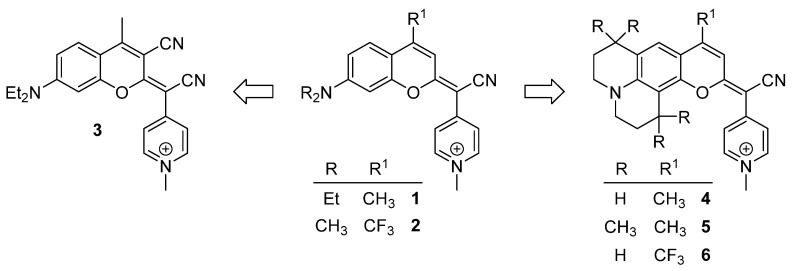
Structure of the original COUPY dyes **1**–**2** and of the analogs **3**–**6** investigated in this work.

In recent years, our group has been a pioneer in describing a new family of fluorophores, named COUPYs, that arise from the incorporation of a cyano(1-alkyl-4-pyridin-1-ium)methylene motif at position 2 of the coumarin backbone [29]. COUPY dyes exhibit orange to deep-red emission, moderate fluorescence quantum yields, large Stokes’ shifts, and excellent cell permeability with preferential accumulation in mitochondria and, to a lesser extent, in nucleoli, being compounds **1** and **2** (Scheme 1), a representative example [29]. In order to optimize the properties of the first-generation COUPY dyes, we have explored several possible modifications of the original molecular framework. The introduction of an azetidine group at position 7 resulted in an increased photostability and an extended Stokes’ shift [30]. A series of analogs where the original *para*-pyridinium moiety was replaced with other electron-deficient heterocycles has also been described. While the *ortho*-pyridinium and the *ortho*,*ortho*-pyrimidinium analogs underwent a blueshift in the absorption and emission maxima, the *ortho*,*para*-pyrimidinium analogs exhibited a redshifted absorption and emission, and higher photostability and selectivity for mitochondria than the parent *para*-pyridinium compound [31]. COUPY dyes have been successfully employed to fluorescently label biomolecules, such as peptides and lipids [32], to develop photosensitizers for anticancer PDT [33,34] and as mitochondria-targeted photolabile protecting groups [35].

Based on these precedents, in this work, we have focused on investigating how the photophysical properties and subcellular localization of COUPY fluorophores can be modulated through the introduction of new modifications in the core scaffold of the parent compounds **1** and **2**. On the one hand, we synthesized compound **3** (Scheme 1) incorporating an EWG at position 3 (i.e., CN) since this strategy has been widely used to redshift absorption and emission maxima in conventional coumarins. On the other hand, the original *N*,*N*-dialkylamino benzene group was replaced with a julolidine (compounds **4** and **6**) or a 1,1,7,7-tetramethyljulolidine (compound **5**) heterocycle, as the fluorescence quantum yield of 7-aminocoumarins is known to be strongly influenced by the substituents on the amino group. It is believed that in this class of fluorescent dyes, especially in polar media, the planar emissive ICT excited state can eventually evolve into a non-fluorescent twisted ICT (TICT) state [36]. However, rigid amino substituents at position 7 have been reported to prevent this twisting process and, as a result, to avoid non-radiative decay attributed to the TICT state [36]. Thus, the introduction of the conformationally restrained fused (1,1,7,7-tetramethyl)julolidine ring was expected to result in an enhancement of fluorescence emission in the new analogs. In addition, a strong EWG was incorporated at position 4 (i.e., CF_3_ in compound **6**) since we envisioned that a higher redshifted absorption and emission would be achieved compared with its 4-methyl counterpart (compound **4**).

Considering all the above mentioned, herein we describe the synthesis of a series of COUPY dyes incorporating modifications in the EDG at position 7 of the coumarin backbone, and/or in the electron density of the pyran ring through the incorporation of EWG (CN or CF_3_) at positions 3 or 4, to evaluate the impact on their photophysical properties and subcellular localization in living HeLa cells.

## 2. Results

### 2.1. Synthesis and Characterization of COUPY Fluorophores ***3***–***6***

**Scheme 2:**
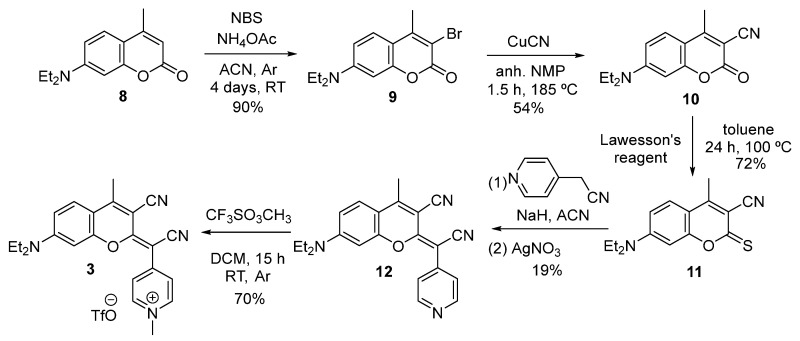
Synthesis of COUPY fluorophore **3**.

The synthesis of COUPY derivative **3** is depicted in Scheme 2. Starting from commercially available coumarin precursor **8**, the regioselective bromination [37], using an equimolar amount of *N*-bromosuccinimide and ammonium acetate, afforded the monobrominated intermediate **9** as a sole product, and in high yields (90%). Subsequently, the cyano group was introduced at position 3 by treatment with cuprous cyanide in *N*-methylpyrrolidone (54%) [28]. Then, following the methodology previously reported by our group for the preparation of COUPY dyes [29], thionation of the lactone carbonyl group in **10** with Lawesson’s reagent (LW) gave the corresponding thiocoumarin intermediate **11** (72%). Further condensation with 4-pyridylacetonitrile in the presence of sodium hydride, followed by treatment with silver nitrate, provided the neutral COUPY scaffold **12** in modest yields (19%). Finally, the target compound **3** was obtained as the pure pyridinium triflate salt (70%) by means of an *N*-alkylation of the pyridine nitrogen atom with methyl triflate in DCM.

**Scheme 3:**
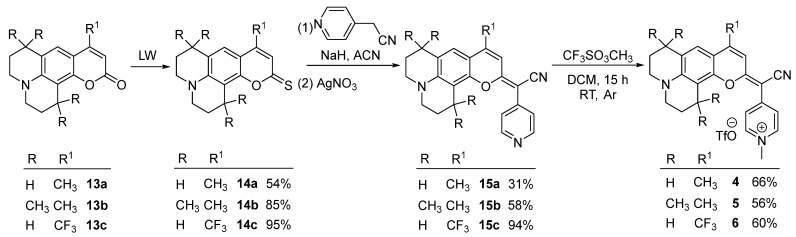
Synthesis of COUPY fluorophore **4**–**6**.

Following the same strategy, the synthesis of COUPY derivatives **4** [34], 5, and **6** [33] was completed from the corresponding commercially available coumarin precursors **13a**–**c** (Scheme 3). In this way, the base-promoted condensation of thiocoumarin intermediates **14a–c** (54–95%) with 4-pyridylacetonitrile led to the advanced intermediates **15a**–**c** (31–94%) upon treatment with AgNO_3_. Once more, *N*-alkylation of the corresponding neutral pyridines with methyl triflate yielded the desired *N*-methylpyridinium COUPY dyes **4**–**6** (56–66%).

All the compounds were fully characterized by ^1^H and ^13^C NMR spectroscopy (Figures S8–S15) and high-resolution ESI mass spectrometry. The purity of the target compounds was assessed by reversed-phase high-performance liquid chromatography (HPLC), obtaining a single peak in all cases (Figure S1). All the chemical characterization data are included in the supplementary materials.

Interestingly, the ^1^H NMR spectra of the neutral COUPY precursors **12** and **15a**–**c** revealed two sets of proton signals that were attributed to the existence of two species in equilibrium in solution resulting from the slow rotation around the exocyclic C=C bond between position 2 of the coumarin scaffold and the 4-pyridylacetonitrile moiety. Due to the strong push–pull effect between the 7-*N*,*N*-dialkylamino group (donor) and the 4-pyridylacetonitrile moiety (acceptor), there is a significant π-delocalization in the system that confers this C=C bond with a partial single bond character, which explains the existence of interconverting *E* and *Z* rotamers rather than diastereomers [29]. The detection of the diagnostic NOE cross-peak between the H*_8_* of the coumarin scaffold and the H*_meta_* of the pyridine ring in the 2D NOESY spectrum of compound **12** (Figure S2) confirmed that the *E*-rotamer was the preferred one (*E*/*Z*: ~80:20 by ^1^H NMR). Similar results were obtained for compounds **15a** and **15c** (*E*/*Z*: ~90:10 in both cases). These results are in agreement with those of structurally related compounds previously reported by our group [29,30,31]. Conversely, in the case of the analog **15b**, the main rotamer was the *Z*-rotamer (*E*/*Z*: ~35:65), as evidenced by the observation of a NOE cross-peak between the H*_3_* of the coumarin scaffold and the H*_meta_* of the pyridine ring in the 2D NOESY spectrum (Figure S3). The different behavior of compound **15b** compared to the other analogs was attributed to the steric effect of the methyl groups in the julolidine moiety, which might favor the prevalence of the *Z*-rotamer. Consistent with our previous reports [29], in the ^1^H NMR spectra of the *N*-methylated COUPY dyes **3**–**6,** we only detected one set of signals, suggesting that a single isomer was found in solution, namely the *E*-isomer for compounds **3**, **4,** and **6** or the *Z*-isomer for compound **5**. The configuration of the main isomer for compounds **3**–**6** could unambiguously be assigned by 2D NOESY experiments (Figures S4–S7).

### 2.2. Photophysical Characterization of COUPY Derivatives

**Figure 2:**
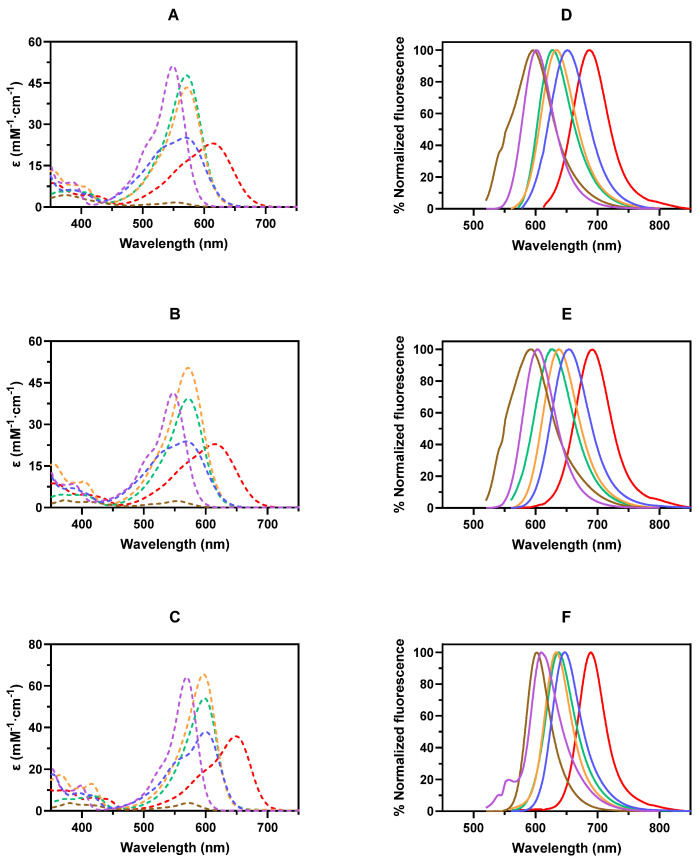
Absorption (dotted lines, panels **A**–**C**) and fluorescence emission (solid lines, panels **D**–**F**) spectra of COUPY fluorophores **1**–**6** in MeOH (top panels), ACN (middle panels), and DCM (bottom panels). Legend: COUPY **1** (purple **–**), **2** (blue **–**), **3** (brown **–**), **4** (orange **–**), **5** (green **–**), and **6** (red **–**).

After completing the synthesis of COUPY dyes **3**–**6**, we investigated their photophysical properties, that is, their absorption and emission spectra, molar absorption coefficients (ε), and fluorescence quantum yields (Φ_F_), in three organic solvents of different polarities and hydrogen bonding abilities (MeOH, ACN, and DCM), and compared them to those of the parent compounds **1** and **2**. The UV–Vis absorption and fluorescence emission spectra of compounds **1**–**6** are shown in Figure 2, and their photophysical properties are summarized in Table 1.

The absorption spectra of COUPY fluorophores **3**–**6** were characterized by the presence of an intense band in the visible region of the electromagnetic spectrum, with the absorption maxima ranging from 553 nm (**3**) to 613 nm (**6**) in MeOH. As previously reported for compounds **1** and **2** [29], the absorption spectra of COUPY dyes **3**–**6** also showed a negative solvatochromism, i.e., a blue shift in the absorption maxima in solvents with higher polarity (e.g., for compound **6**, the *λ*_abs_ shifted from 652 nm in DCM to 613 nm in MeOH), and a significant hyperchromism in non-polar solvents (DCM). The introduction of structural modifications in the COUPY scaffold had a great influence on the absorption properties of the resulting compounds. On the one hand, the replacement of the *N*,*N*-dialkylamino group at position 7 by the julolidine-fused ring in analogs **4**–**6** resulted in a redshift in their absorption maxima (e.g., the *λ*_abs_ of compounds **1** and **4** in MeOH were 549 and 572 nm, respectively). On the other hand, as described for the parent compounds **1** and **2** [29], the replacement of the CH_3_ group at position 4 by an electron-withdrawing CF_3_ group induced a strong bathochromic effect in the absorption maximum of compound **6** (i.e., the *λ*_abs_ of compounds **4** and **6** in MeOH were 572 and 613 nm, respectively). Notably, both 4-CF_3_-containing dyes **2** and **6** exhibited wider absorption bands and lower molar absorption coefficients (*ε*) than their 4-CH_3_ counterparts (e.g., *ε* = 43.4 and 23.1 mM^−1^ cm^−1^ for **4** and **6**, respectively, in MeOH). Surprisingly, the introduction of an electron-withdrawing cyano group at position 3 had very little influence on the location of the absorption maxima, but it caused a dramatic reduction in the *ε* of the resulting COUPY dye **3** (*ε* = 1.6 mM^−1^ cm^−1^ in MeOH).

The chemical modification of the coumarin backbone not only affected the absorption spectra of the respective COUPY dyes but also had a deep impact on their emissive properties. As shown in Figure 2, the emission spectra of COUPY dyes **3**–**6** presented an intense band with the maximum wavelength ranging from 595 nm (**3**) to 685 nm (**6**) in MeOH. In general, the emission maxima of the compounds exhibited a slight negative solvatochromism, and the fluorescent quantum yields (Φ_F_) were significantly enhanced in non-polar solvents (DCM) in all cases (Table 1). The COUPY derivatives incorporating a julolidine-fused ring (**4**–**6**) displayed a redshift in the emission band when compared to their *N*,*N*-dialkyl counterparts. Interestingly, while compounds **4** and **5** displayed very similar molar absorption coefficients (*ε* = 43.4 and 47.8 mM^−1^ cm^−1^ for **4** and **5**, respectively, in MeOH), the Φ_F_ of compound **4** (Φ_F_ = 0.14, in MeOH) was much higher than that of compound **5** (Φ_F_ = 0.01, in MeOH). The introduction of the 4-CF_3_ group in analog **6** caused a noticeable shift to longer wavelengths in its emission spectrum (e.g., *λ*_abs_ of compounds **4** and **6** in MeOH were 633 and 685 nm, respectively) and a considerable reduction in its Φ_F_ values in contrast to the corresponding 4-CH_3_ analog **4** (Φ_F_ = 0.14 and 0.03 for **4** and **6**, respectively, in MeOH). Surprisingly, the 3-cyano analog **3** showed a very weak fluorescence (Φ_F_ < 0.01, in MeOH), and the emission maxima were blueshifted with respect to that of the parent compound **1**.

Finally, the photostability of COUPY dyes **1**–**6** in water (Figures S16 and S17) was assessed under yellow LED light (560 ± 40 nm, 40 mW/cm^2^) irradiation. As shown in Figure S17, all the tested dyes were considerably stable up to light fluences of 24 J/cm^2^. Notably, at the experiment endpoint, none of the studied compounds, other than analog **4**, underwent photobleaching greater than 40%. The lower extent of photodegradation of fluorophores **2** and **6**, compared to that of **1** and **4**, indicates that the incorporation of a trifluoromethyl group at position 4 of the COUPY scaffold not only shifts the absorption and the emission maxima to longer wavelengths but also results in an enhancement of the photostability.

### 2.3. Confocal Microscopy Studies

**Figure 3:**
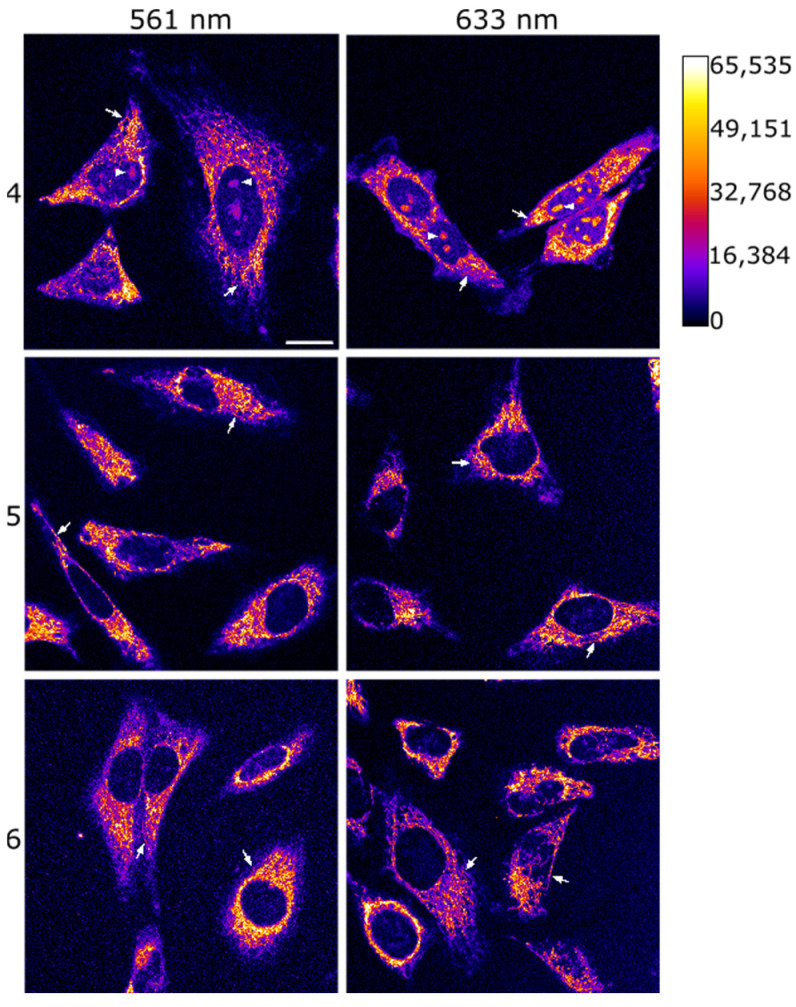
Cellular uptake of COUPY dyes **4**, **5,** and **6**. Single confocal planes of HeLa cells treated for 30 min at 37 °C with compounds **4** (top), **5** (middle), or **6** (bottom) at a final concentration of 2 μM after excitation at 561 nm (left panels) or 633 nm (right panels). Fluorescent signal is shown in Fire LUT; the calibration bar is shown in the top right corner of the figure. White arrows point out mitochondria, and white arrowheads in the top row point out nucleoli. Scale bar: 20 μm; all images are at the same scale.

After confirming the promising photophysical features of the julolidine-containing COUPY dyes **4**–**6**, we next investigated the potential of these compounds for imaging applications in living cells. First, confocal microscopy was used to evaluate their cellular uptake in HeLa cells upon irradiation with either a yellow-light laser (λ_ex_ = 561 nm) or a red-light laser (λ_ex_ = 633 nm). Gratifyingly, after only 30 min of incubation at a concentration of 2 µM (37 °C), a clear fluorescence signal was detected inside the cells (Figure 3, Figures S20 and S21), demonstrating that the three fluorophores were efficiently internalized. Additionally, no noticeable cell toxicity was observed across the different COUPY dyes. This finding is in line with the fact that the reported IC_50_ values for some of these compounds [33,34] are significantly higher than the concentrations utilized in this study. All three compounds displayed a clear filamentous staining pattern, suggesting mitochondrial accumulation [40]. Interestingly, while compound **4** exhibited a greater value for the Φ_F_ than compound **5** (see above section), the mean intensity of the fluorescence signal detected for **5** was much higher than that of **4** (Figure S18). Furthermore, while compound **5** almost exclusively stained mitochondria, COUPY dye **4** was also detected in nucleoli (Figure S19), which is consistent with the staining pattern of previously reported COUPY dyes [29]. These observations suggest that the presence of the methyl groups in the julolidine moiety of compound **5** might affect the lipophilicity of the COUPY molecule, thus resulting in an enhanced cellular uptake and a more specific mitochondrial accumulation.

**Figure 4:**
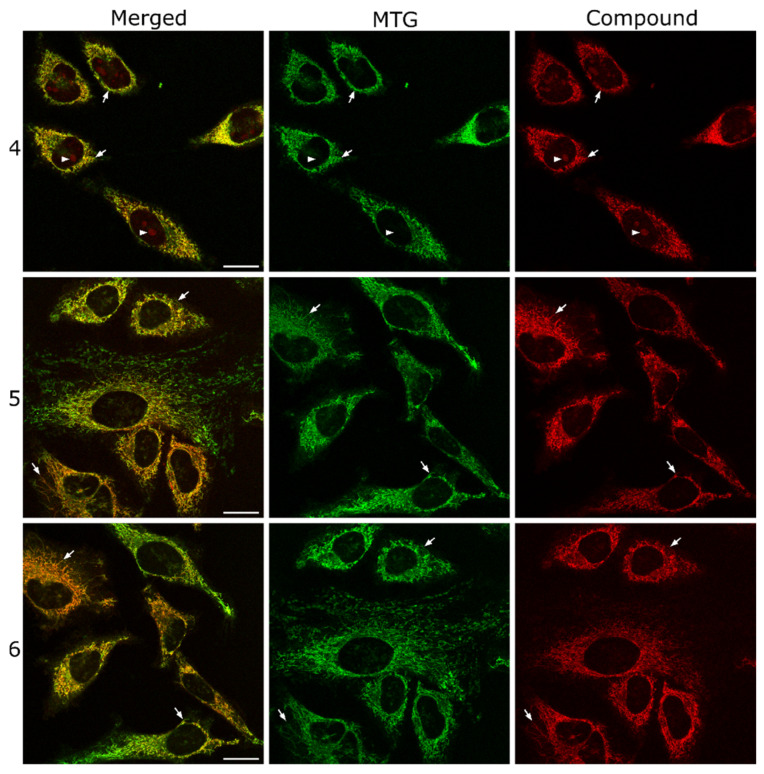
Colocalization studies of COUPY dyes **4** (top), **5** (middle), and **6** (bottom) with MitoTracker Green FM (MTG). Single confocal planes of HeLa cells incubated with the corresponding coumarin dye (2 μM, λ_Ex_ = 561 nm, λ_Em_ = 575–650 nm, red) and MTG (1 μM, λ_Ex_ = 488 nm, λ_Em_ = 500–550 nm, green) both for 30 min at 37 °C. Left: merged images; center: MTG signal; right: compound signal. White arrows point out some colocalizing mitochondria, and white arrowheads in the top row point out nucleoli. Scale bar: 20 μm; all images are at the same scale.

In order to confirm the subcellular localization of COUPY dyes **4**–**6**, a series of co-localization experiments were conducted using the mitochondria-specific fluorescent marker MitoTracker Green FM (MTG). As depicted in Figure 4 and Figure S22, a striking overlap was found between the fluorescence signals of **4** and MTG, suggesting a significant degree of colocalization between the dye and the mitochondria. Moreover, the calculated Pearson’s correlation coefficient (PCC = 0.80) and the Manders’ colocalization coefficients (M1 = 0.73; M2 = 0.77) further confirmed this observation, indicating a robust and statistically significant association. The high PCC value signifies a strong linear relationship between the intensities of the dye and MTG, whereas the Manders’ coefficients emphasize the proportion of each fluorophore’s signal overlapping with the other. Similarly, colocalization experiments between **5** or **6** and MTG (Figure 4, Table S1) revealed a strong correlation between the staining pattern of the COUPY dyes and that of MTG, leading to comparable results for the calculated coefficients (**5**: PCC = 0.81, M1 = 0.77; M2 = 0.75; **6**: PCC = 0.77, M1 = 0.69, M2 = 0.77). These results are not surprising since cationic and lipophilic compounds have a well-documented capacity for targeting mitochondria, owing to the negative potential across the outer and inner mitochondrial membrane [41,42].

Finally, to better understand the nature of the other subcellular compartments stained by COUPY **4**, we also performed a colocalization experiment between compound **4** and Hoechst 33342, which is a fluorescent probe typically used to visualize DNA in live cells. Even though Hoechst efficiently stains the nucleus, it does not target the nucleoli, which are dense structures within the nucleus where ribosomal RNA (rRNA) is transcribed and assembled with proteins to form ribosomal subunits. As depicted in Figure S19, the absence of overlap between the structures stained by **4** within the nucleus and the fluorescence signal of Hoechst 33342 provided indirect evidence of the partial accumulation of **4** in nucleoli.

## 3. Discussion

To summarize, in this study, we have explored the impact of different structural modifications of the coumarin backbone on the photophysical properties and subcellular localization of the resulting COUPY dyes in comparison with parent compounds **1** and **2**. On the one hand, in COUPY dyes **3** and **6**, an EWG was incorporated at position 3 or 4, respectively, to increase the push–pull effect. On the other hand, in dyes **4**–**6**, the 7-*N*,*N*-dialkylamino group was replaced by a more rigid julolidine-fused ring aiming to reduce non-radiative decay processes. COUPY dyes **3**–**6** were prepared from inexpensive commercially available precursors following a common synthetic strategy in which the key step was the condensation reaction of a thiocoumarin intermediate with 4-pyridylacetonitrile, followed by the *N*-methylation of the pyridine nitrogen atom. We studied the photophysical properties of the dyes and compared them with those of **1** and **2**. In this way, we were able to establish a series of structure–photophysical property relationships: (1) the introduction of EWG at position 3 caused a dramatic reduction of the *ε* and Φ_F_ values of the 3-cyano derivative **3** and, hence, it should be avoided for COUPY dyes; (2) the replacement of the 7-*N*,*N*-dialkylamino group by the julolidine-fused ring lead to a significant bathochromic shift in the absorption and emission maxima of dyes **4**–**6**, which also displayed excellent *ε* values and moderate Φ_F_ values, comparable to those of the parent compounds; (3) the introduction of a trifluoromethyl group at position 4 shifted the emission maxima of the resulting COUPY dye **6** toward the NIR region and resulted in a substantial enhancement in the photostability. Julolidine-containing COUPY dyes **4**–**6** were efficiently internalized in living HeLa cells, and colocalization studies with MTG demonstrated their preferential accumulation in the mitochondria. Overall, our findings offer valuable insights for the rational design of new mitochondria-targeted coumarin-based dyes with deep-red/NIR emission. Work is currently in progress in our laboratory to explore the bathochromic effect of other structural modifications in the COUPY scaffold and to exploit the mitochondria-targeting ability of COUPY dyes for theragnostic applications.

## 4. Materials and Methods

### 4.1. General Remarks

Unless specified otherwise, common chemicals and solvents of HPLC-grade or reagent-grade quality were purchased from commercial sources and used without additional purification. For all reactions requiring heat, a hot plate magnetic stirrer, accompanied by an aluminum reaction block of the appropriate size, served as the heating source. Thin-layer chromatography analyses (TLC) were carried out on aluminum plates coated with a 0.2 mm thick layer of silica gel 60 F254. Column chromatography purification was performed using silica gel 60 (230–400 mesh). Reversed-phase high-performance liquid chromatography (HPLC) analyses were conducted with a Waters Alliance 2695 Separations Module featuring a quaternary pump solvent delivery module, online degasser, autosampler, and a Waters 2996 photodiode array detector. For HPLC separation, a Jupiter Proteo C12 column (50 mm × 4.6 mm, 90 Å, 4 µm) from Phenomenex was used. The mobile phase followed a linear gradient from 90:10 (*v*/*v*) A/B to 0:100 (*v*/*v*) A/B over 4.5 min at a flow rate of 1.5 mL/min (A: 0.1% formic acid in H_2_O; B: 0.1% formic acid in ACN). The injection volume was 10 µL. Control of the HPLC instrument and processing of the chromatogram output, including annotation of retention times, integration of peaks, and calculation of peak areas, were performed using MassLynx V4.1 software. NMR spectra were recorded at 25 °C in a 400 MHz Bruker Avance Neo spectrometer using the deuterated solvent as an internal deuterium lock. The residual protic signal of the deuterated solvent served as a reference in ^1^H and ^13^C{^1^H} NMR spectra. Chemical shifts are reported in parts per million (ppm) in the δ scale, coupling constants in Hz, and multiplicity is indicated as follows: s (singlet), d (doublet), t (triplet), q (quartet), qt (quintuplet), m (multiplet), dd (doublet of doublets), dq (doublet of quartets), br (broad signal), etc. Proton signals of the *E* and *Z* rotamers were identified through a simple inspection of the ^1^H spectrum, and the rotamer ratio was calculated by peak integration. Two-dimensional NOESY spectra (2D-NOESY) were acquired in CDCl_3_ or DMSO-*d*_6_ with mixing times of 500 ms. High-resolution electrospray ionization mass spectra (ESI-HRMS) were recorded on a G1969A LC/MSD-TOF instrument from Agilent Technologies (Santa Clara, CA, USA).

### 4.2. Synthetic Procedures

COUPY fluorophores **4** and **6** were synthesized following previously reported procedures [33,34].

#### 4.2.1. Synthesis of COUPY Fluorophore **3**

##### Compound **9**

A solution of *N*-bromosuccinimide (11.43 g, 64.2 mmol) in acetonitrile (150 mL) was added dropwise to a solution of coumarin **8** (15.0 g, 64.2 mmol), and ammonium acetate (4.95 g, 64.2 mmol) in acetonitrile (100 mL) under an argon atmosphere. The reaction mixture was stirred for 4 days at room temperature. After removing the solvent under reduced pressure, H_2_O (200 mL) was added, and the crude mixture was extracted with ethyl acetate (3 × 100 mL). The combined organic layers were washed with H_2_O (3 × 100 mL), washed with saturated NaCl (100 mL), dried over anhydrous MgSO_4,_ and filtered. The solvent was removed under vacuum to give 17.85 g (90% yield) of a brown solid, which was used without further purification in the next step. TLC: R_f_ (DCM) 0.38; ^1^H NMR (400 MHz, CDCl_3_) δ (ppm): 7.42 (1H, d, *J* = 9.2 Hz), 6.60 (1H, dd, *J* = 9.2, 2.8 Hz), 6.49 (1H, d, *J* = 2.8 Hz), 3.41 (4H, q, *J* = 7.2 Hz), 2.53 (3H, s), 1.21 (6H, t, *J* = 7.2 Hz); ^13^C{^1^H} NMR (101 MHz, CDCl_3_) δ (ppm): 158.2, 154.5, 151.6, 150.7, 126.17, 109.1, 109.0, 105.7, 97.3, 44.9, 19.2, 12.5; HRMS (ESI-TOF) (*m*/*z*): [M+H]^+^ calcd for C_14_H_17_BrNO_2_, 310.0437; found, 310.0430.

##### Compound **10**

Coumarin **9** (1.36 g, 4.42 mmol) and CuCN (0.61 g, 6.84 mmol) were dissolved in dry NMP (4 mL) under an argon atmosphere. The reaction mixture was stirred for 3 h at 185 °C in the dark. After bringing the reaction mixture to room temperature, acetone was added, the crude was filtered, and the resulting solution was evaporated under vacuum. The product was dissolved in ethyl acetate (100 mL) and washed with water (3 × 50 mL). The organic layer was dried over magnesium sulfate and filtered. The solvent was removed under vacuum. After purification by column chromatography (silica gel, 0–70% ethyl acetate in hexanes), 0.70 g (54% yield) of a brown solid was obtained. TLC: R_f_ (EtOAc/hexanes 1:1) 0.46; ^1^H NMR (400 MHz, CDCl_3_) δ (ppm): 7.47 (1H, d, *J* = 9.2 Hz), 6.65 (1H, dd, *J* = 9.2, 2.4 Hz), 6.45 (1H, d, *J* = 2.4 Hz), 3.46 (4H, q, *J* = 7.2 Hz), 2.62 (3H, s), 1.24 (6H, *J* = 7.2 Hz); ^13^C{^1^H} NMR (101 MHz, CDCl_3_) δ (ppm): 161.4, 158.8, 156.5, 153.1, 127.5, 115.3, 109.9, 107.7, 97.3, 93.7, 45.2, 17.7, 12.5; HRMS (ESI-TOF) (*m*/*z*): [M+H]^+^ calcd for C_15_H_17_N_2_O_2_, 257.1285; found, 257.1279.

##### Compound **11**

Coumarin **10** (0.58 g, 2.26 mmol) and Lawesson’s reagent (0.95 g, 2.35 mmol) were dissolved in toluene (60 mL) and heated at 100 °C for 24 h and protected from light. After evaporation under reduced pressure, the dark residue was purified by column chromatography (silica gel, 50–90% DCM in hexanes) to give 0.44 g of an orange solid (yield 72%). TLC: R_f_ (DCM) 0.40; ^1^H NMR (400 MHz, CDCl_3_) δ (ppm): 7.49 (1H, d, *J* = 9.2 Hz), 6.71 (1H, dd, *J* = 9.2, 2.4 Hz), 6.59 (1H, d, *J* = 2.4 Hz), 3.47 (4H, q, *J* = 7.2 Hz), 2.58 (3H, s), 1.25 (6H, *J* = 7.2 Hz); ^13^C{^1^H} NMR (101 MHz, CDCl_3_) δ (ppm): 192.7, 158.7, 153.8, 153.3, 127.6, 116.2, 111.2, 109.8, 108.3, 97.1, 45.4, 17.5, 12.6; HRMS (ESI-TOF) (*m*/*z*): [M+H]^+^ calcd for C_15_H_17_N_2_OS, 273.1062; found, 273.1062.

##### Compound **12**

Coumarin **11** (400 mg, 1.47 mmol), 4-pyridylacetonitrile (521 mg, 4.4 mmol), and sodium hydride (60% dispersion in mineral oil, 1.0 g, 25 mmol) were dissolved in dry acetonitrile (20 mL) under an argon atmosphere and protected from light. After stirring for 3 h at 40 °C, silver nitrate (600 g, 3.53 mmol) was added, and the reaction mixture was stirred at room temperature for 3 h under an argon atmosphere and protected from light. The crude product was evaporated under reduced pressure and purified by column chromatography (silica gel, 0–100% ethyl acetate in DCM, and then 0–5% MeOH in ethyl acetate) to give 100 mg of an orange/brown solid (yield 19%). TLC: R_f_ (EtOAc/DCM 3:1) 0.40; ^1^H NMR (400 MHz, CDCl_3_) δ (ppm): (major rotamer) 8.65 (2H, m), 7.88 (2H, d, *J* = 6.4 Hz), 7.48 (1H, d, *J* = 9.2 Hz), 6.66 (1H, dd, *J* = 9.2 Hz, *J* = 2.4 Hz), 6.30 (1H, d, *J* = 2.4 Hz), 3.50 (4H, q, *J* = 7.2 Hz), 2.66 (3H, s), 1.26 (6H, t, *J* = 7.2 Hz); ^13^C{^1^H} NMR (101 MHz, CDCl_3_) δ (ppm): (major rotamer) 159.7, 157.3, 154.5, 153, 7, 145.4, 127.8, 125.1, 122.8, 116.9, 114.1, 110.8, 108.9, 96.2, 94.7, 82.9, 45.4, 17.9, 12.6; HRMS (ESI-TOF) (*m*/*z*): [M+H]^+^ calcd for C_22_H_21_N_4_O, 357.1710; found, 357.1715.

##### Compound **3**

Methyl triflate (31 µL, 0.27 mmol) was added to a solution of coumarin **12** (20 mg, 0.055 mmol) in DCM (10 mL). The mixture was stirred overnight at room temperature under an argon atmosphere and protected from light. After evaporation under reduced pressure and purification, 20 mg of a dark purple solid (yield 70%) was obtained. TLC: R_f_ (10% MeOH in DCM) 0.35; ^1^H NMR (400 MHz, CDCl_3_) δ (ppm): 8.63 (2H, d, *J* = 7.2 Hz), 8.25 (2H, d, *J* = 7.2 Hz), 7.57 (1H, d, *J* = 9.6 Hz), 7.03 (1H, d, *J* = 2.4 Hz), 6.79 (1H, dd, *J* = 9.6 Hz, *J* = 2.4 Hz), 4.26 (3H, s), 3.63 (4H, q, *J* = 7.2 Hz), 2.73 (3H, s), 1.29 (6H, t, *J* = 7.2 Hz); ^13^C{^1^H} NMR (101 MHz, DMSO-*d_6_*) δ (ppm): 163.2, 160.1, 154.5, 154.1, 149.3, 144.2, 128.9, 122.6, 116.7, 114.2, 112.4, 109.4, 95.9, 92.8, 78.3, 46.4, 44.6, 17.8, 12.4; HRMS (ESI-TOF) (*m*/*z*): [M]^+^ calcd for C_23_H_23_N_4_O, 371.1866; found, 371.1869; analytical HPLC (10 to 100% B in 4.5 min): R_t_ = 2.68 min.

#### 4.2.2. Synthesis of COUPY Fluorophore **5**

##### Compound **14b**

Lawesson’s reagent (7.79 g, 19.27 mmol) was added to a solution of coumarin **13b** (10 g, 32.11 mmol) in toluene (250 mL). The resulting solution was stirred under reflux at 100 °C overnight and protected from light. The mixture was allowed to cool to room temperature, and the solvent was removed under reduced pressure. The crude product was purified by column chromatography (silica gel, 0–100% DCM in hexanes) to give 8.94 g of an orange solid (85% yield). TLC: R_f_ (DCM) 0.65; ^1^H NMR (400 MHz, CDCl_3_) δ (ppm): 7.27 (1H, s), 6.90 (1H, s), 3.37–3.30 (2H, m), 3.28–3.20 (2H, m), 2.29 (3H, s), 1.85–1.80 (2H, m), 1.78–1.73 (2H, m), 1.60 (6H, s), 1.31 (6H, s); ^13^C{^1^H} NMR (101 MHz, CDCl_3_) δ (ppm): 195.7, 155.3, 146.6, 145.9, 129.7, 123.3, 119.6, 114.8, 111.7, 47.4, 47.0, 39.5, 35.7, 32.6, 32.4, 30.7, 29.2, 18.3; HRMS (ESI): *m*/*z* calcd. for C_20_H_26_NOS [M+H]^+^ 328.1730; found 328.1731.

##### Compound **15b**

Coumarin **14b** (3 g, 9.19 mmol), sodium hydride (60% dispersion in mineral oil, 1.1 g, 45.8 mmol), and 4-pyridylacetonitrile hydrochloride (2.12 g, 13.74 mmol) were dissolved in anhydrous acetonitrile (500 mL) under an argon atmosphere in the dark. After stirring for 4 h at room temperature, AgNO_3_ (3.42 g, 20.15 mmol) was added, and the reaction mixture was stirred for 2 h under an argon atmosphere and protected from light. Then, the solvent was removed under reduced pressure, and the crude product was purified by column chromatography (silica gel, 0–2.5% MeOH in DCM) to give 1.70 g of a brown solid (45% yield). TLC: R_f_ (10% MeOH in DCM): 0.73; ^1^H NMR (400 MHz, CDCl_3_) δ (ppm): (major rotamer *E*) 8.56 (2H, m), 7.38 (2H, m), 7.13 (1H, s), 6.52 (1H, s), 3.30 (2H, m), 3.18 (2H, m), 2.26 (3H, s), 1.85 (2H, m), 1.76 (2H, m), 1.66 (6H, s), 1.30 (6H, s); ^13^C{^1^H} NMR (101 MHz, CDCl_3_) δ (ppm): (rotamers *E* + *Z*) 163.5, 163.1, 151.0, 150.1, 149.9, 145.8, 145.1, 144.6, 143.9, 142.7, 141.4, 128.1, 123.9, 122.9, 120.7, 119.8, 119.7, 116.0, 111.8, 110.6, 108.3, 79.9, 78.8, 47.5, 46.9, 46.5, 40.3, 39.7, 36.1, 35.9, 32.5, 32.0, 31.0, 30.8, 28.9, 28.8, 19.2, 18.9; HRMS (ESI): *m*/*z* calcd. for C_27_H_30_N_3_O [M+H]^+^ 412.2383; found 412.2381.

##### Compound **5**

Methyl triflate (61 μL, 0,54 mmol) was added to a solution of compound **15b** (14.8 mg, 0.04 mmol) in DCM (20 mL). The mixture was stirred overnight at room temperature under an argon atmosphere and protected from light. Then, the solvent was removed under reduced pressure, and the crude product was purified by column chromatography (silica gel, 0–4% MeOH in DCM) to give 11.6 mg of a dark purple solid (56% yield). TLC: R_f_ (10% MeOH in DCM): 0.37; ^1^H NMR (400 MHz, CDCl_3_) δ (ppm): 8.26 (2H, d, *J* = 7.5 Hz), 7.89 (2H, d, *J* = 7.5 Hz), 7.38 (1H, s), 7.19 (1H, s), 4.17 (3H, s), 3.45 (2H, m), 3.34 (2H, m), 2.59 (3H, s), 1.87 (2H, m), 1.78 (2H, m), 1.64 (6H, s), 1.34 (6H, s); ^13^C{^1^H} NMR (101 MHz, CDCl_3_) δ (ppm): 167.0, 154.6, 152.6, 151.9, 148.1, 142.5, 131.8, 121.0, 120.3, 119.4, 114.9, 112.7, 109.1, 47.8, 47.0, 46.4, 39.4, 35.0, 32.7, 32.2, 30.1, 28.6, 19.6; HRMS (ESI): *m*/*z* calcd. for C_28_H_32_N_3_O^+^ [M]^+^ 426.2540; found 426.2546; analytical HPLC (10 to 100% B in 4.5 min): R_t_ = 3.22 min.

## Figures and Tables

**Table 1:** Photophysical data of COUPY fluorophores **1**–**6** in different solvents. ^a^ Wavelength of the absorption maximum; ^b^ Molar absorption coefficient at λ_max_; ^c^ Wavelength of the emission maximum upon excitation at the wavelength shown in parentheses; ^d^ Stokes’ shift (nm); ^e^ Fluorescence quantum yields, measured as described by Resch-Genger and Rurack [38] using Cresyl Violet in MeOH (Φ_F_ = 0.55) [39] as reference.

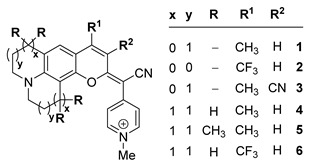						
Compound	Solvent	λ_abs_^a^	ε(mM^−1^cm^−1^) ^b^	λ_em_ (λ_exc_) ^c^	Stokes’ Shift ^d^	Φ_F_^e^
**1**	MeOH	549	51.2	602 (546)	53	0.23
	ACN	548	41.3	603 (546)	55	0.20
	DCM	570	64.2	603 (546)	33	0.70
**2**	MeOH	570	25.2	651 (578)	81	0.02
	ACN	568	23.9	654 (578)	86	0.01
	DCM	601	37.9	646 (578)	45	0.05
**3**	MeOH	553	1.6	595 (546)	42	<0.01
	ACN	554	2.6	592 (546)	38	<0.01
	DCM	573	3.8	604 (546)	31	0.01
**4**	MeOH	572	43.4	633 (578)	61	0.14
	ACN	572	50.4	638 (578)	66	0.15
	DCM	598	65.4	633 (578)	35	0.59
**5**	MeOH	572	47.8	627 (578)	55	0.01
	ACN	572	39.3	628 (578)	56	<0.01
	DCM	599	54.0	637 (578)	38	0.04
**6**	MeOH	613	23.1	685 (578)	72	0.03
	ACN	614	22.9	690 (578)	76	0.03
	DCM	652	35.7	690 (578)	38	0.13

## Data Availability

Data are contained in the article and Supplementary Materials or will be provided by the authors.

## Supplementary Materials

The following supporting information can be downloaded at: https://www.mdpi.com/article/10.3390/ijms242417427/s1, References [43,44,45,46,47] are cited in the supplementary materials.
