# Supplementary material for: Exploring Structural–Photophysical Property Relationships in Mitochondria-Targeted Deep-Red/NIR-Emitting Coumarins
Source: Int J Mol Sci. 2023 Dec 13;24(24):17427. doi: 10.3390/ijms242417427 (PMC10743691; doi:10.3390/ijms242417427)
Supplement: Supplementary file 1 [file ijms-24-17427-s001.zip › ijms-2725964-supplementary.pdf]

SUPPLEMENTARY MATERIAL

Exploring Structural–Photophysical Property Relationships in Mitochondria-Targeted Deep-Red/NIR-Emitting Coumarins

Eduardo Izquierdo-García [1], Anna Rovira [1], Joan Forcadell [1], Manel Bosch [2], Vicente Marchán [1,*]

[1] Secció de Química Orgànica, Departament de Química Inorgànica i Orgànica, Institut de Biomedicina de la Universitat de Barcelona (IBUB), Universitat de Barcelona (UB), Carrer Martí i Franquès 1–11, E-08028 Barcelona, Spain

[2] Unitat de Microscòpia Òptica Avançada, Centres Científics i Tecnològics de la Universitat de Barcelona (CCiTUB), Universitat de Barcelona (UB), Avinguda Diagonal 643, E-08028 Barcelona, Spain

* Correspondence: vmarchan@ub.edu

Table of contents

1.- Reversed-phase HPLC analysis of COUPY fluorophores …..….…. S3

2.- 2D NMR characterization of COUPY derivatives ………………….. S4

3.- ^{1}H and ^{13}C NMR spectra of the compounds ………………………… S10

4.- Photophysical studies …………..…………….……………………. S18

5.- Confocal microscopy studies……………………...………………….. S21

6.- References……………………………………...………………… S26

1. Reversed-phase HPLC analysis of COUPY fluorophores

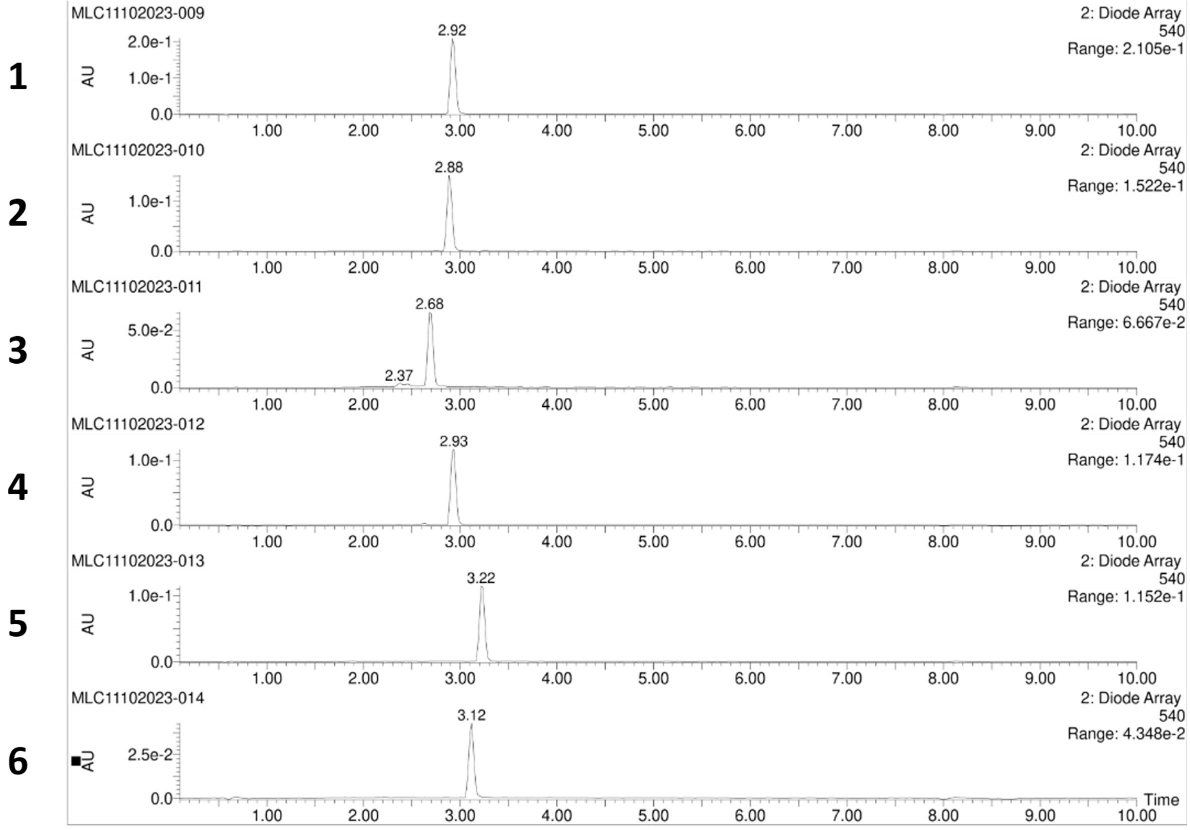

Figure S1. Reversed-phase HPLC traces of COUPY dyes **1-6**.

2. 2D NMR characterization of COUPY derivatives

Compound 12

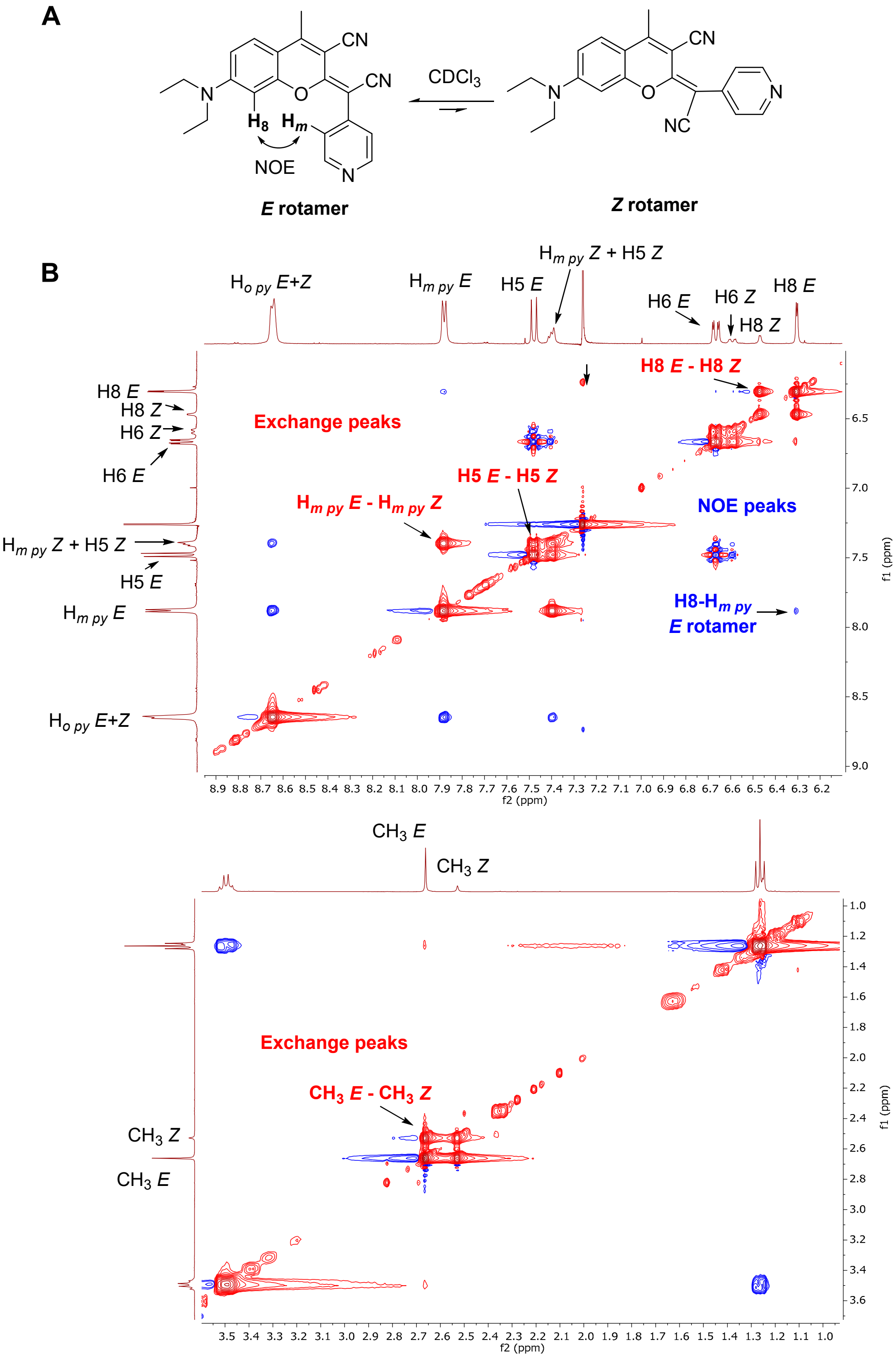

Figure S2. Structure of E and Z rotamers of coumarin **12** with some diagnostic NOE cross-peaks indicated, and expansions of the NOESY spectrum (tm = 500 ms) of **12** in DMSO-d6 at 298 K showing exchange cross-peaks between rotamer resonances of the same sign as the diagonal.

Compound 15b

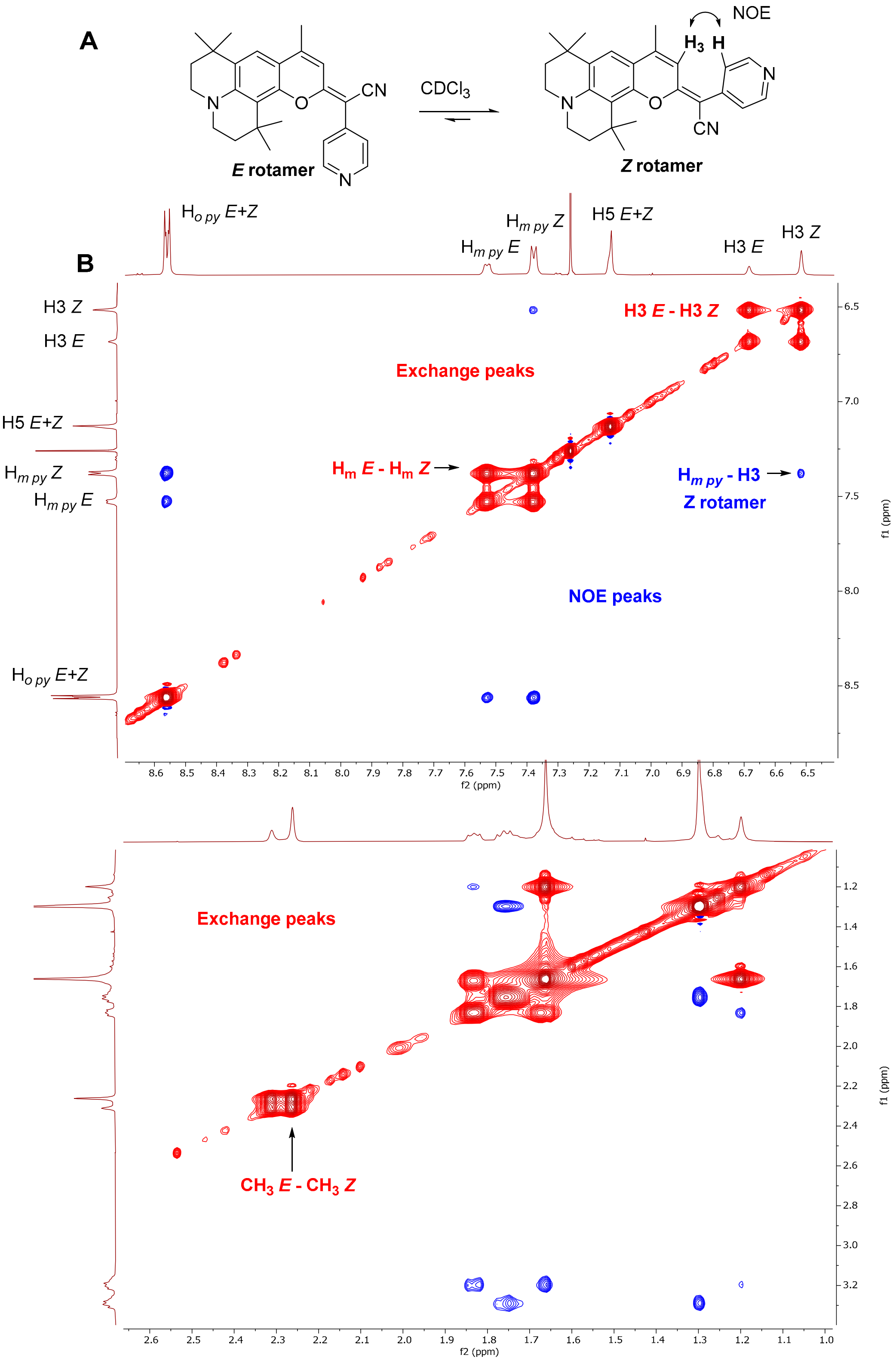

Figure S3. Structure of *E* and *Z* rotamers of coumarin **15b** with some diagnostic NOE cross-peaks indicated, and expansions of the NOESY spectrum (t_m = 500 ms) of **15b** in $CDCl_3$ at 298 K showing exchange cross-peaks between rotamer resonances of the same sign as the diagonal.

Compound 3

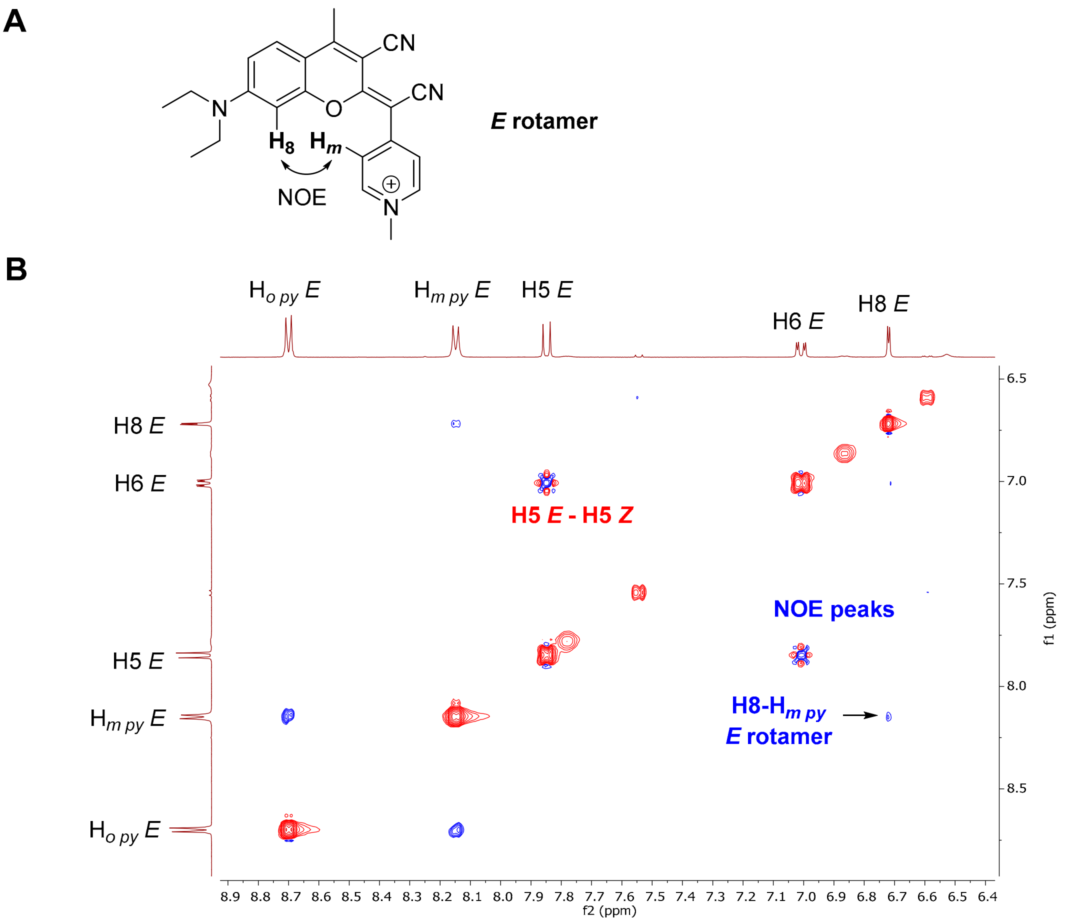

Figure S4. Structure of *E* rotamer of coumarin **3** with some diagnostic NOE cross-peaks indicated (A), and expansions of the NOESY spectrum (t_m = 500 ms) of **3** in DMSO-d_6 at 298 K (B).

Compound 4

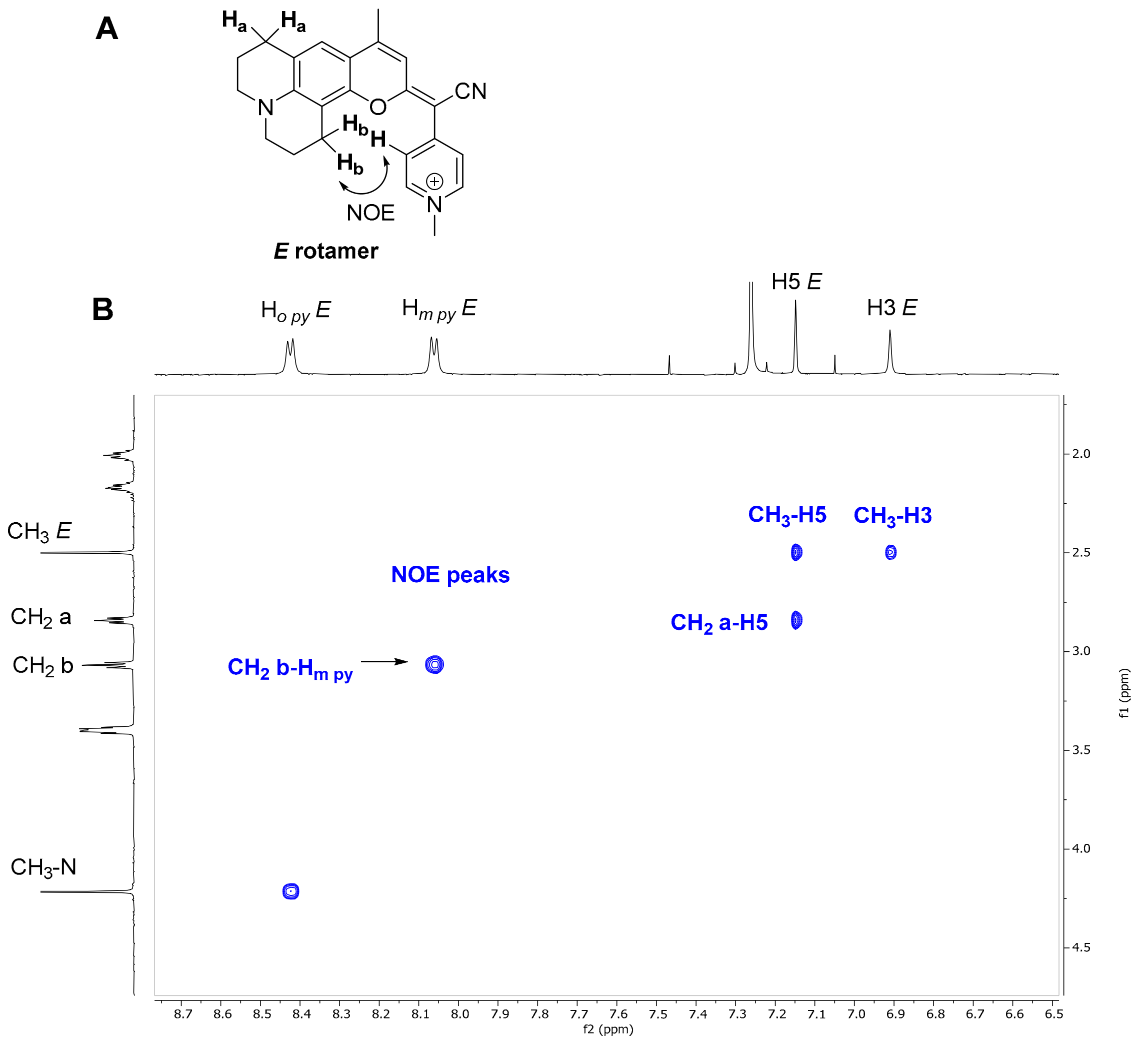

Figure S5. Structure of *E* rotamer of coumarin **4** with some diagnostic NOE cross-peaks indicated, and expansion of the NOESY spectrum (t_m = 500 ms) of **4** in $CDCl_3$ at 298 K.

Compound 5.

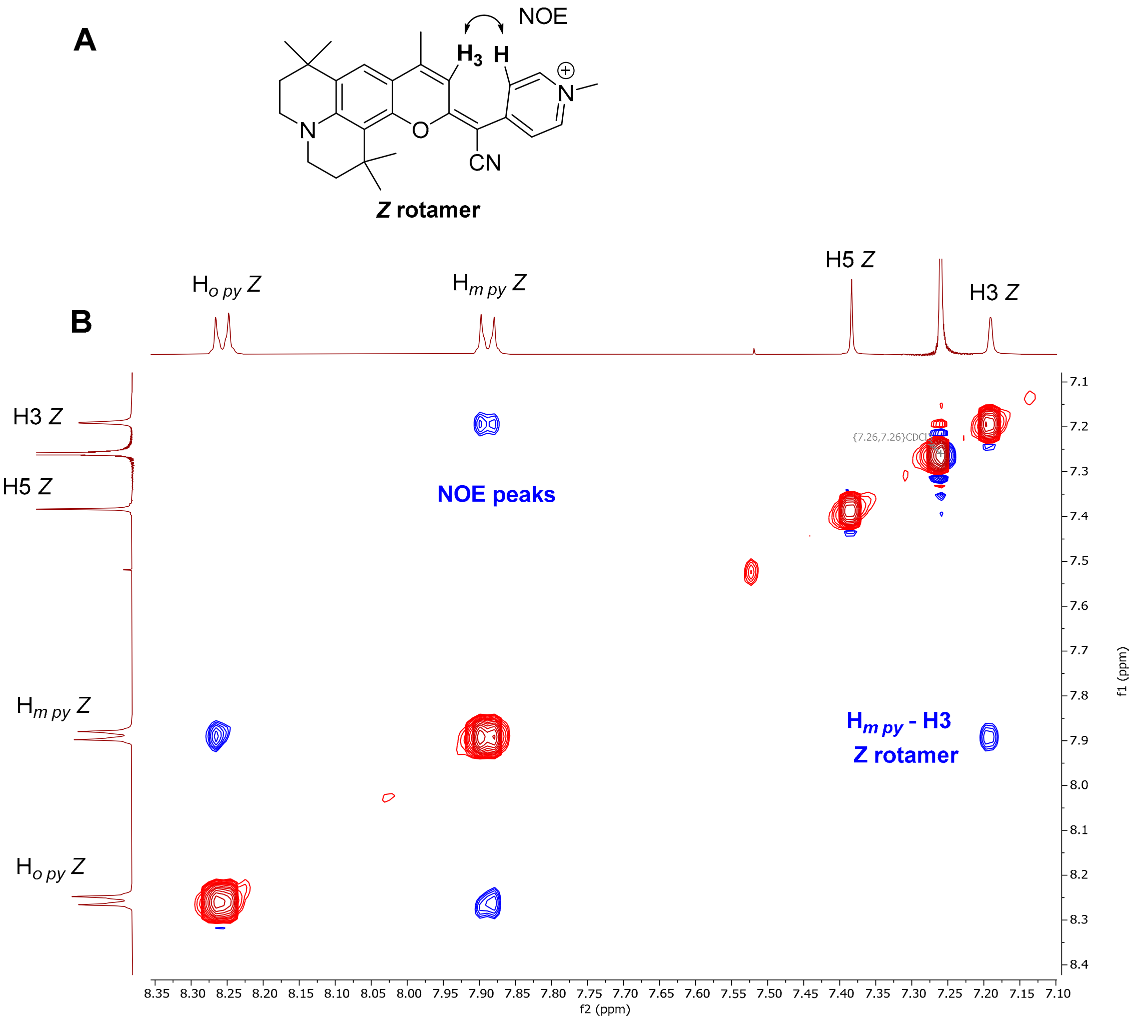

Figure S6. Structure of *Z* rotamer of coumarin **5** with some diagnostic NOE cross-peaks indicated, and expansion of the NOESY spectrum (t_m = 500 ms) of **5** in $CDCl_3$ at 298 K.

Compound 6

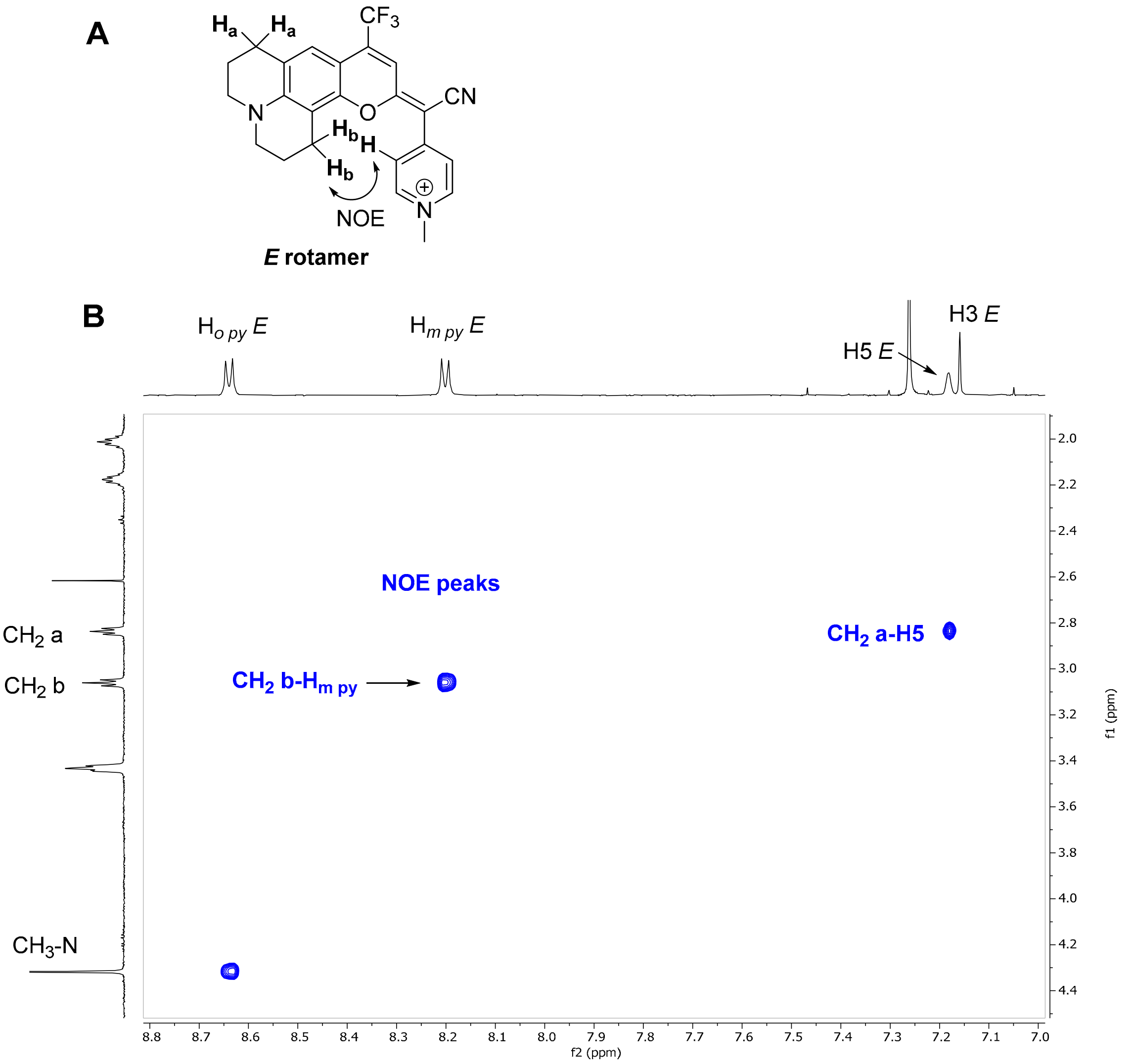

Figure S7. Structure of *E* rotamer of coumarin **6** with some diagnostic NOE cross-peaks indicated, and expansion of the NOESY spectrum (t_m = 500 ms) of **6** in $CDCl_3$ at 298 K.

3. ^{1}H and ^{13}C NMR spectra of the compounds

Compound 9

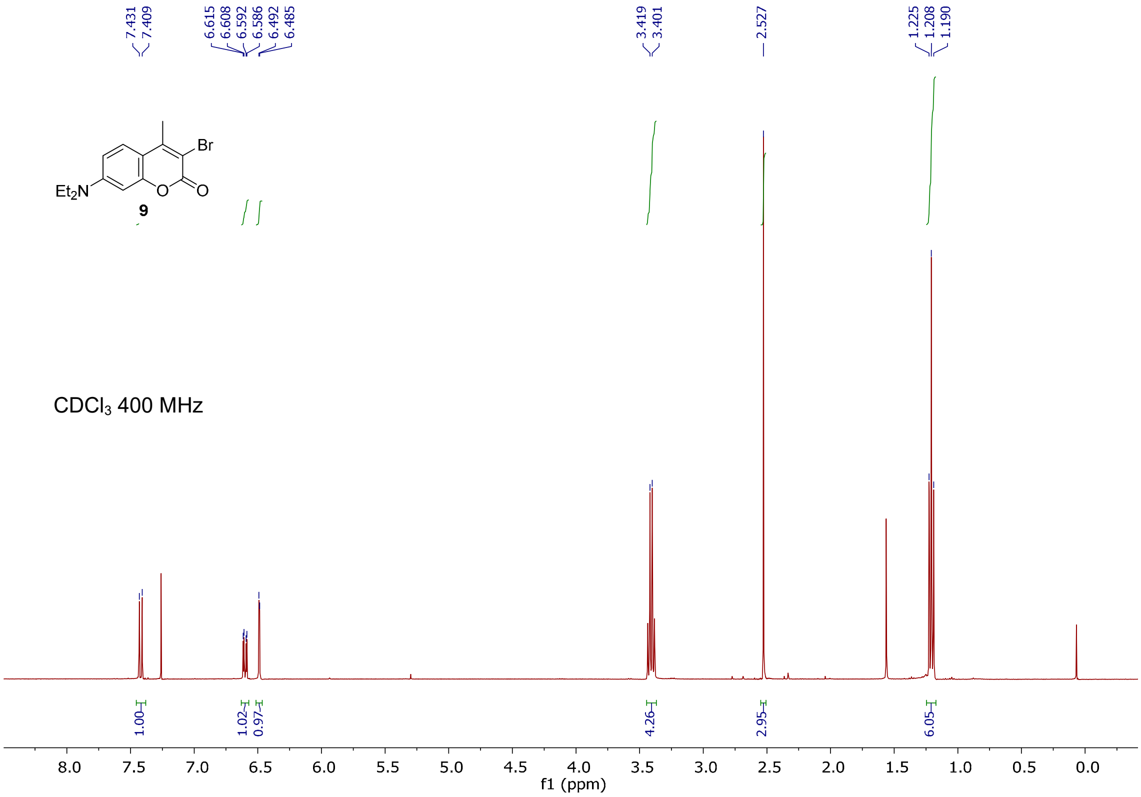

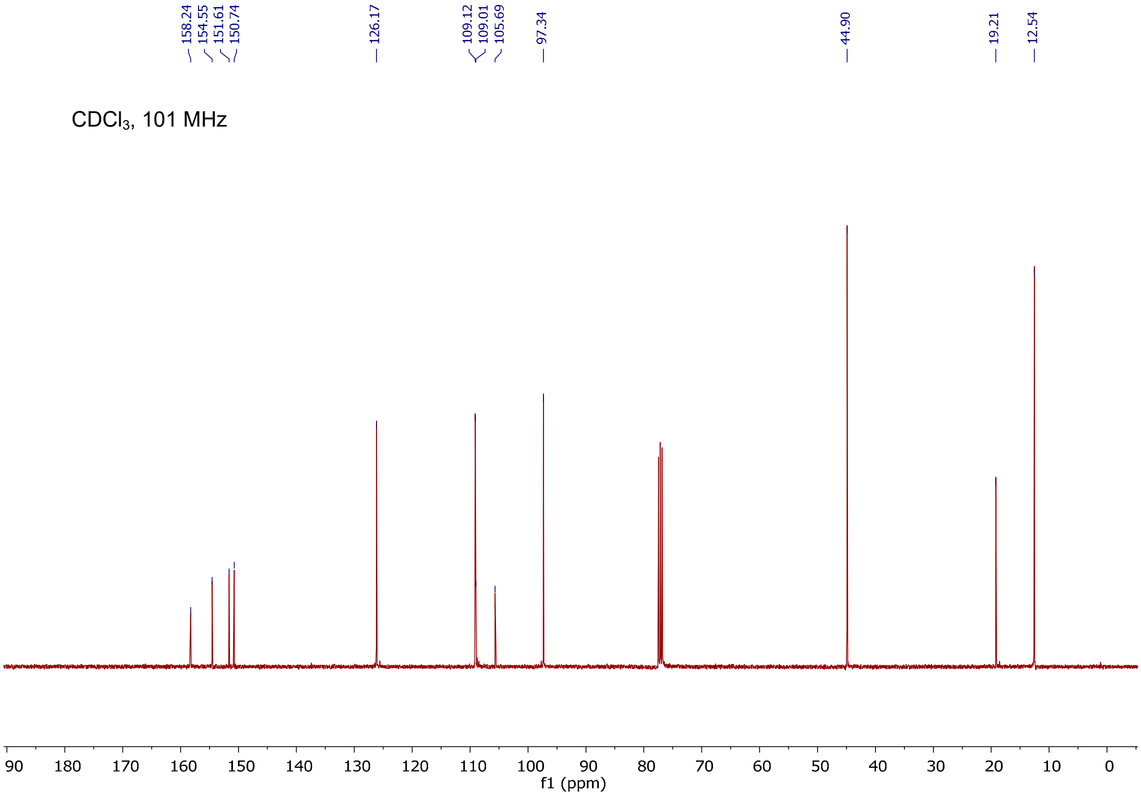

Figure S8. ^{1}H and ^{13}C NMR spectra of compound **9** in $CDCl_3$.

Compound 10

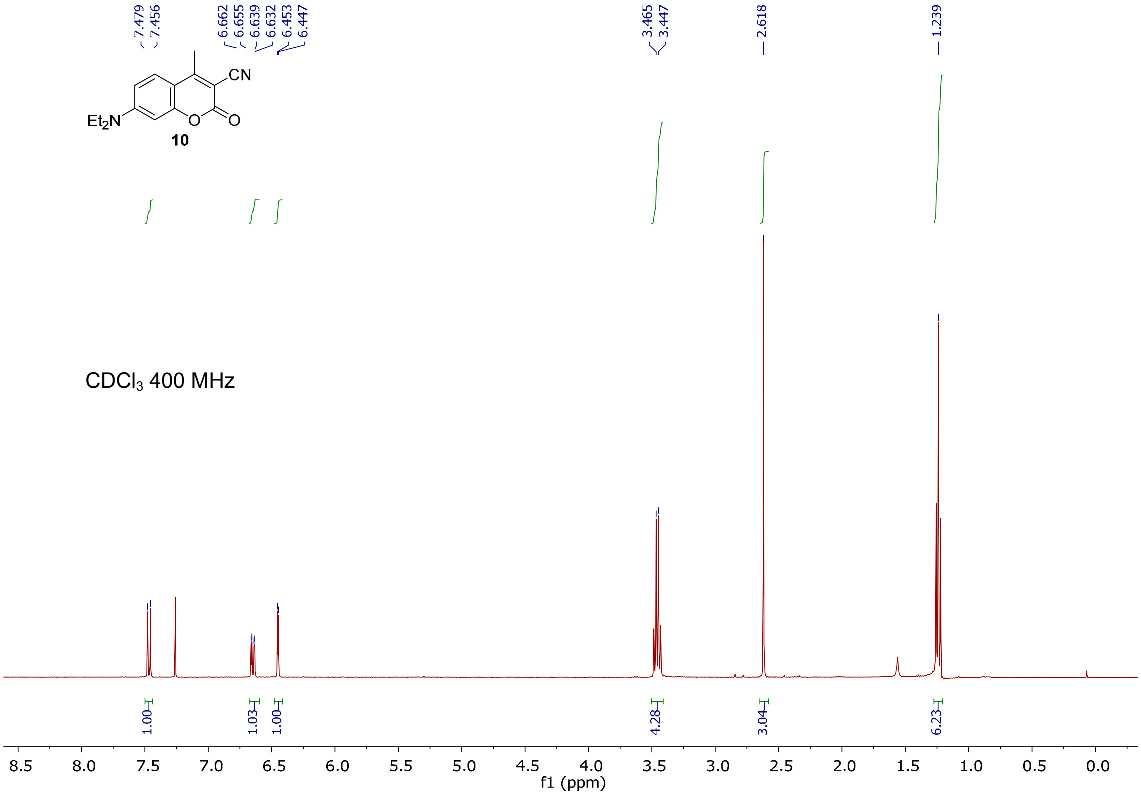

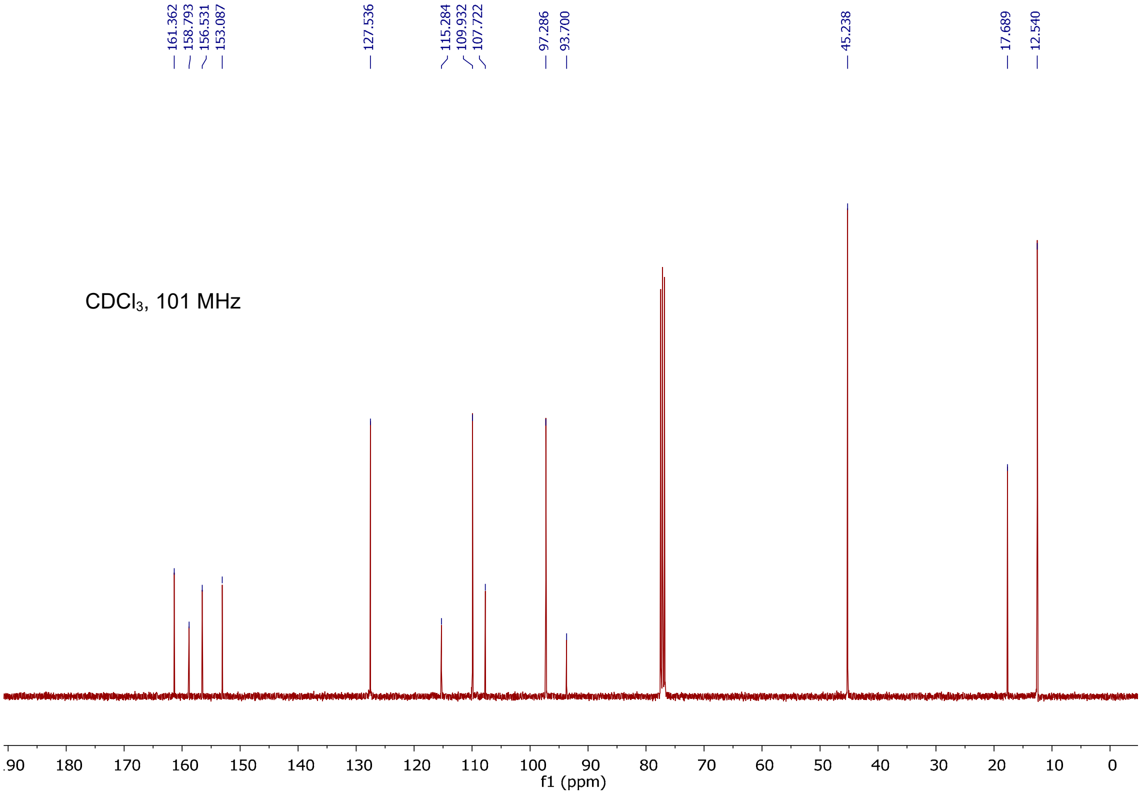

Figure S9. ^{1}H and ^{13}C NMR spectra of compound **10** in $CDCl_3$.

Compound 11

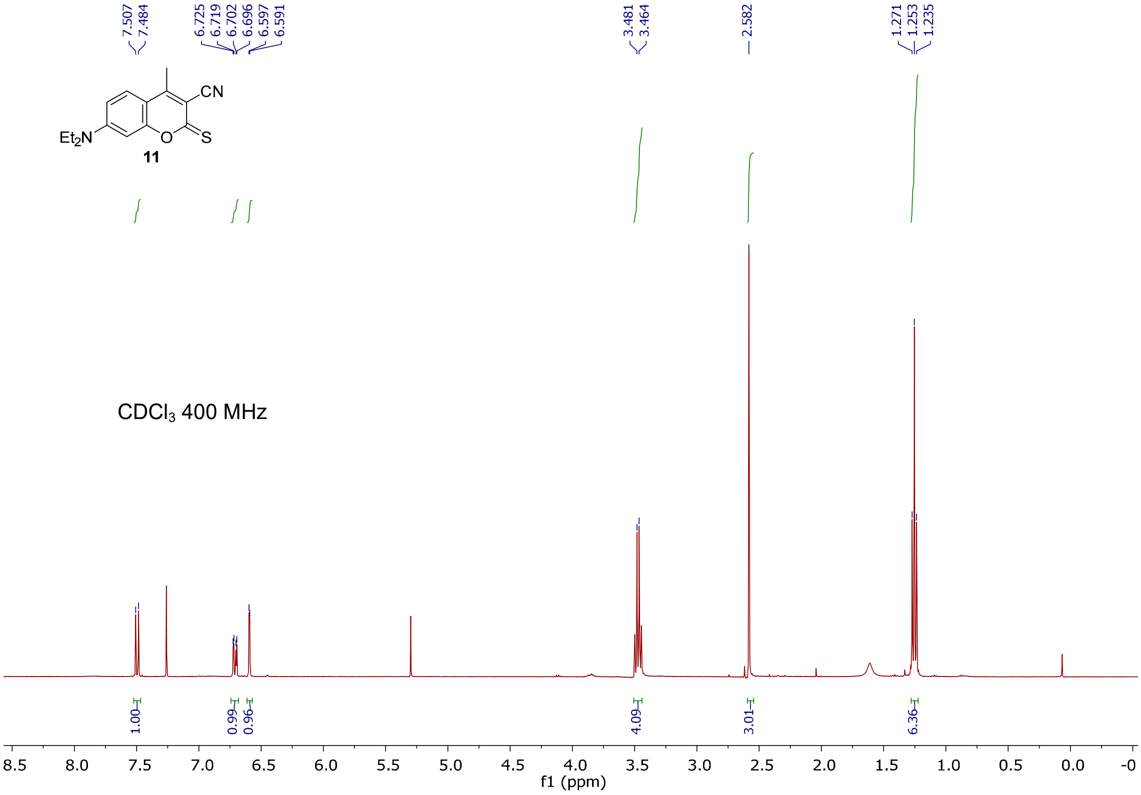

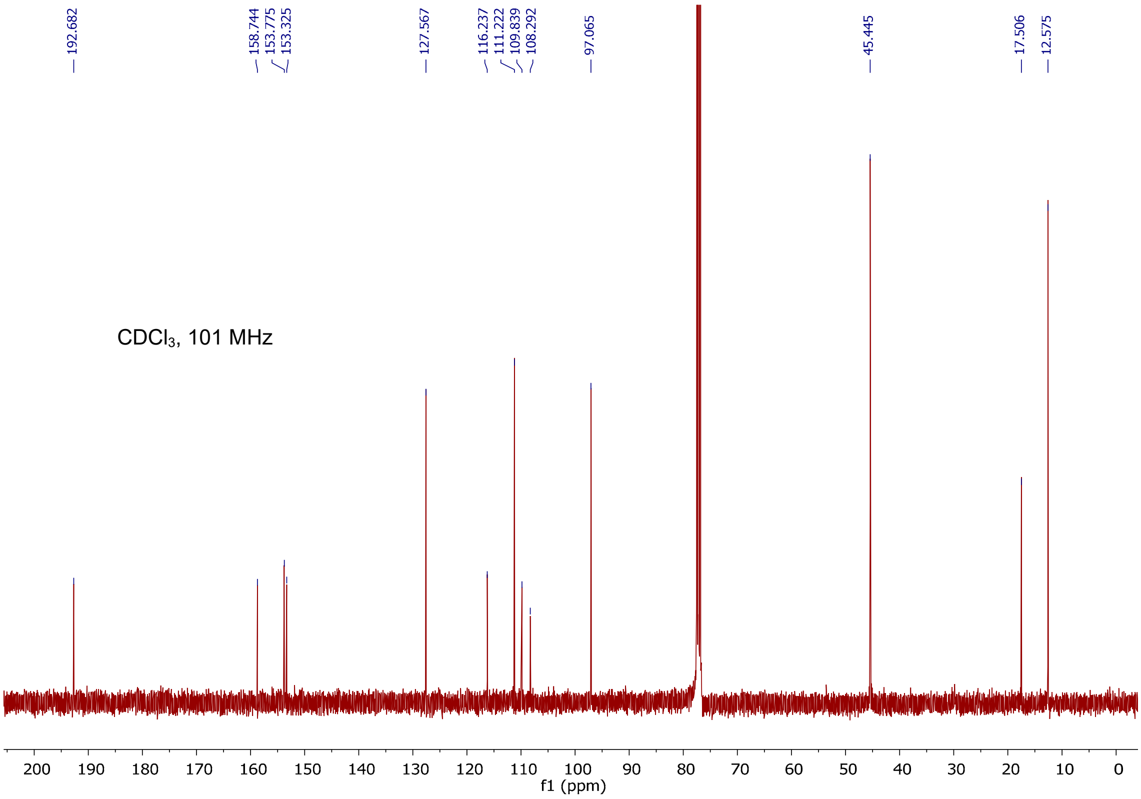

Figure S10. ^{1}H and ^{13}C NMR spectra of compound **11** in $CDCl_3$.

Compound 12

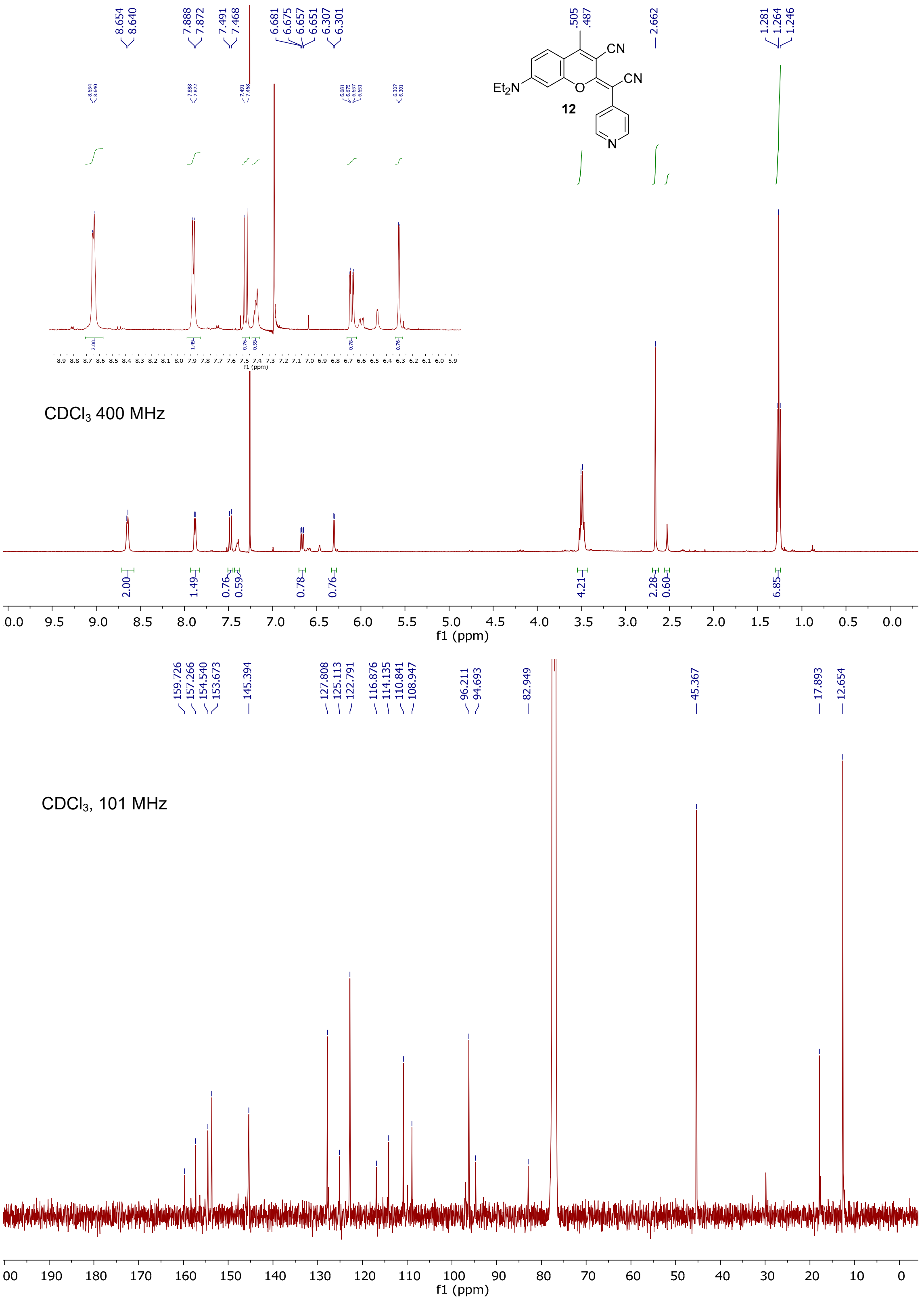

Figure S11. ^{1}H and ^{13}C NMR spectra of compound **12** in $CDCl_3$.

Compound 3

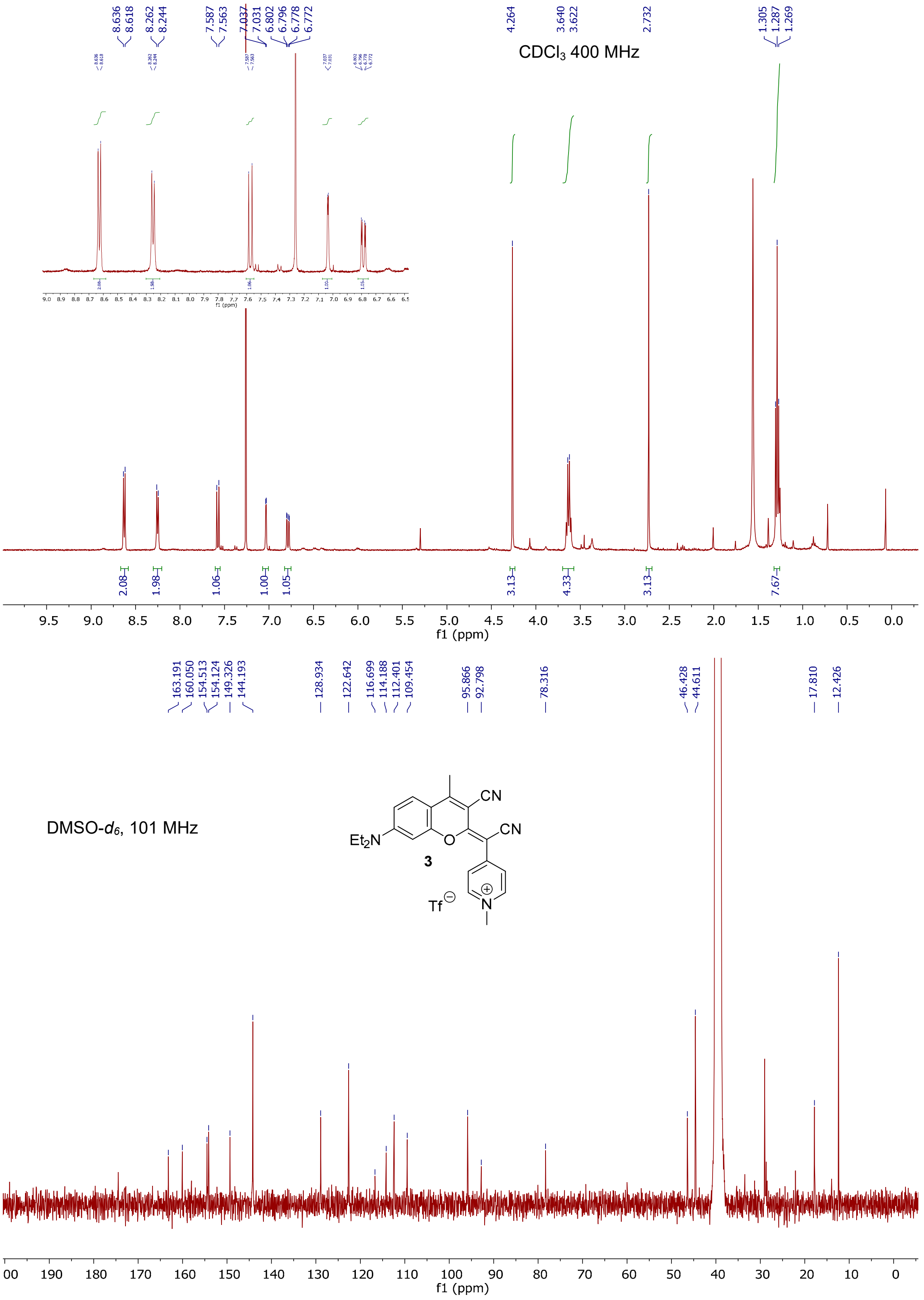

Figure S12. ^{1}H and ^{13}C NMR spectra of compound **3** in $CDCl_3$ and DMSO-d_6, respectively

Compound 14b

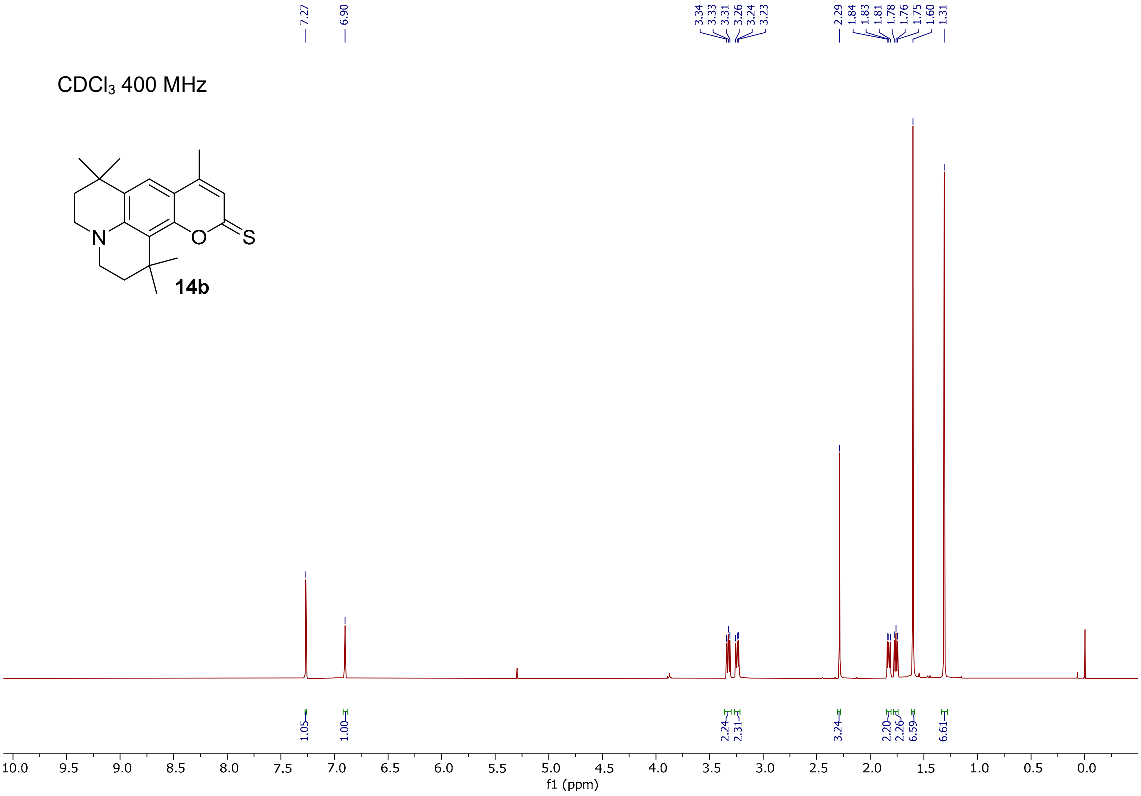

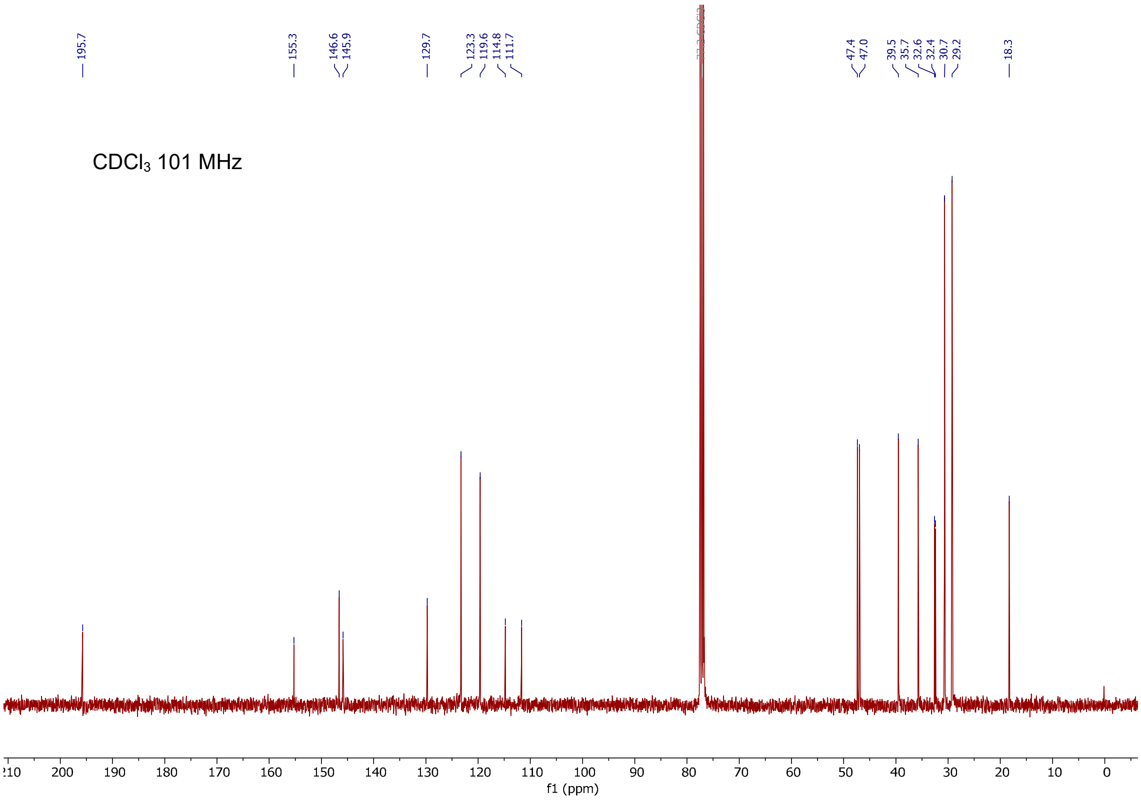

Figure S13. ^{1}H and ^{13}C NMR spectra of compound **14b** in $CDCl_3$.

Compound 15b

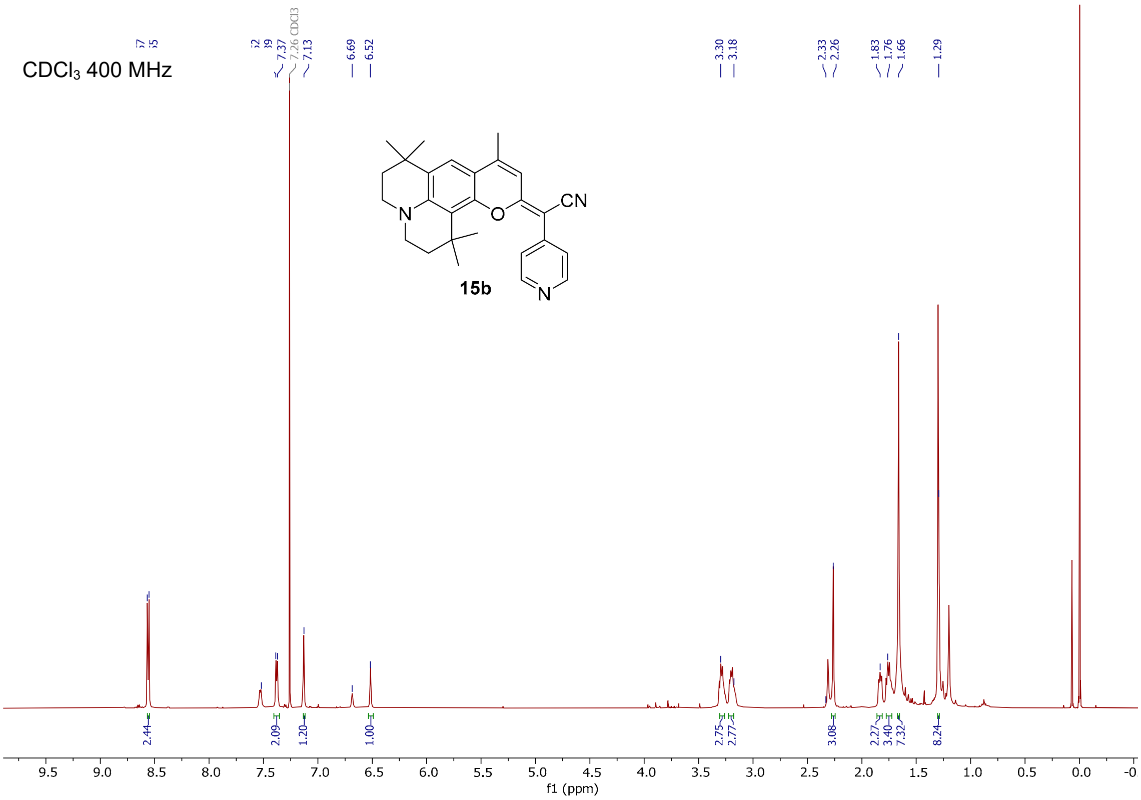

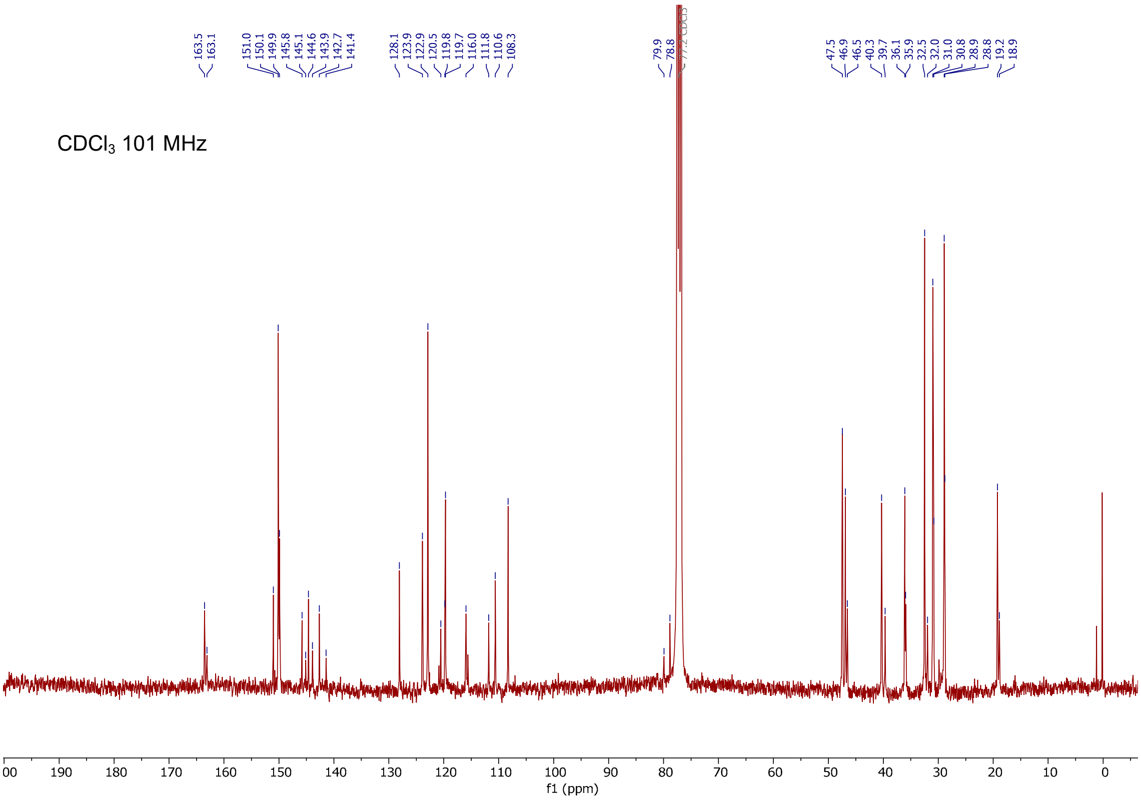

Figure S14. ^{1}H and ^{13}C NMR spectra of compound **15b** in $CDCl_3$.

Compound 5

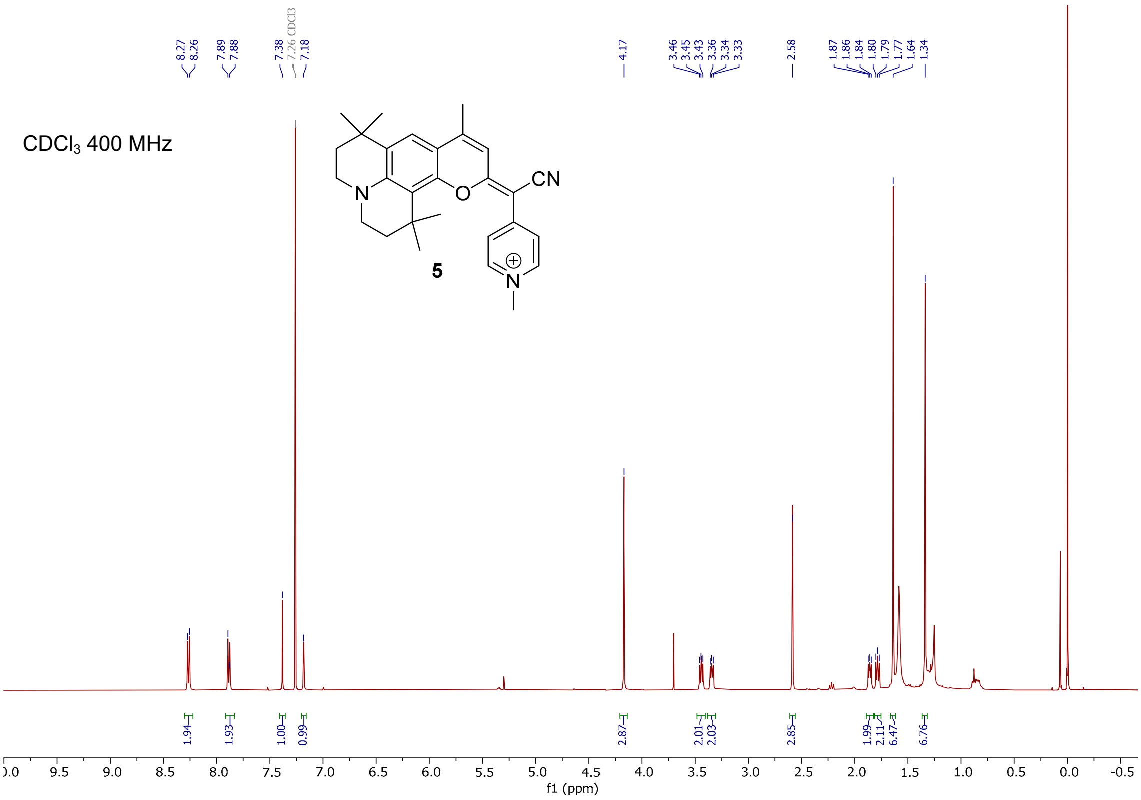

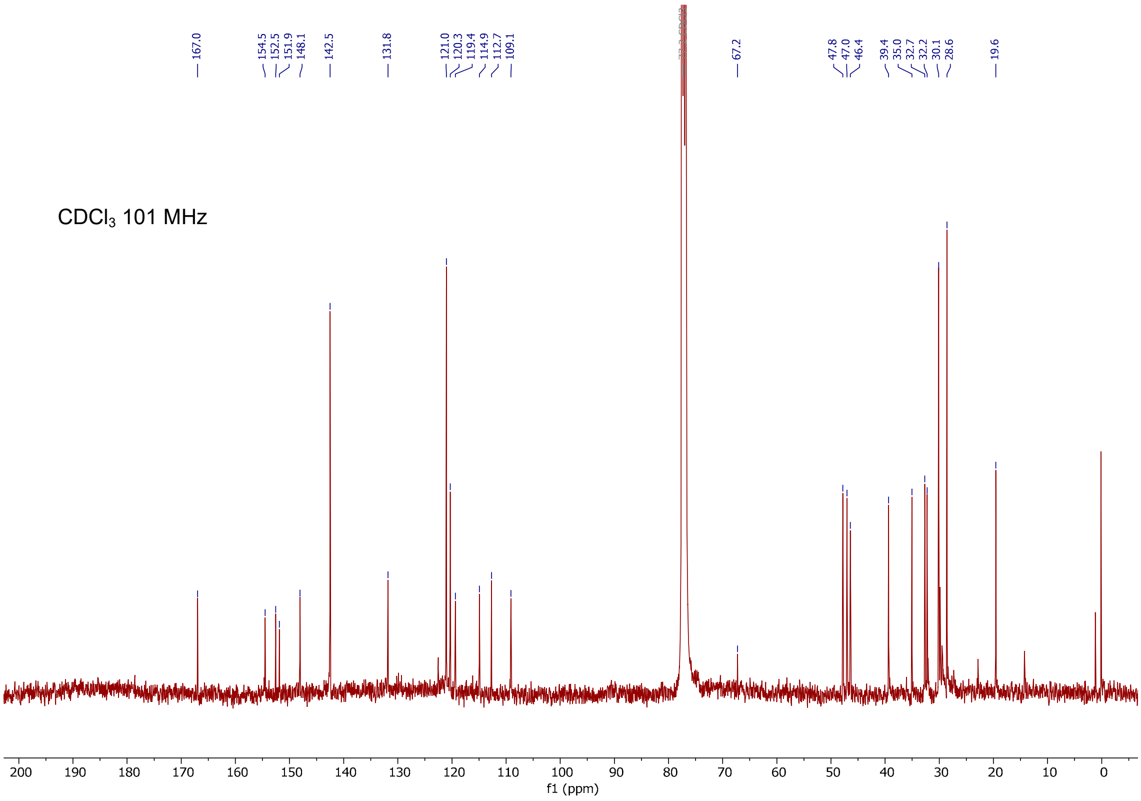

Figure S15. ^{1}H and ^{13}C NMR spectra of compound **5** in CDCl3.

4. Photophysical studies

4.1. Absorption and emission spectra.

Absorption spectra were recorded on a Jasco V-730 UV-Vis spectrophotometer using a spectral bandwidth of 1 nm, a response time of 0.24 sec, a data interval of 1 nm and a scan rate of 200 nm/min. Measurements were carried at a constant temperature of 25 ºC. The temperature was maintained with a MultiTemp III Thermostatic Circulator from Pharmacia Biotech. Fluorescence emission spectra were recorded on a Photon Technology International (PTI) QuantaMaster fluorometer at room temperature. The excitation and emission monochromators were set at 1 mm, giving a spectral bandwidth of 4 nm. The data interval was 1 nm and the integration time was 1 sec. All measurements were carried using a Hellma 1.5 mL PTFE-stoppered fluorescence quartz cuvette with a 1 cm path length.

4.2. Molar extinction coefficient.

The molar extinction coefficients (ε) were calculated according to Lambert-Beer's law, represented in **Equation S1**, where A is absorbance, ε is the molar extinction coefficient, l is the pathlength of the cuvette (cm) and c is the concentration (M).

$$A = \varepsilon \cdot l \cdot c \qquad \textbf{Equation S1}$$

In this way, a series of solutions at concentrations ranging between 1 and 40 μM was prepared in the appropriate solvent system (spectrophotometric grade solvents), and the absorption spectrum of each solution was measured as described above. The absorbance value at the λ_{Abs} was then plotted against the corresponding concentration and adjusted to a linear regression function forced through the origin (*i.e.,* the line was forced to intercept (0,0)) using GraphPad Prism version 9.00 for Windows (GraphPad Software Inc., La Jolla, USA). Only the absorbance values in the range between 0.05 and 1 were used. Since we used a 1 cm path length cuvette, ε equals the slope of the graph.

4.3. Fluorescence quantum yield.

The fluorescence quantum yields (Φ_F) were measured following the comparative method described by Resch-Genger and Rurack [1] (IUPAC technical report). In this way, a series of solutions of the test compounds and the Cresyl violet standard were prepared such that the *Abs* value at the corresponding λ_{Ex} was approximately between 0.01 and 0.1. The absorption and emission spectra of each solution were recorded using a 1 cm path length quartz cuvette, as described above. The test compounds and the standard were analysed using the same settings.

The integrated fluorescence intensity (*i.e.* the area under the curve of the emission spectrum) was plotted against the corresponding Abs value at the λ_{Ex} and adjusted to a linear regression function using GraphPad Prism version 9.00 for Windows (GraphPad Software Inc., La Jolla, CA, USA). Then, Φ_F was calculated using **Equation S2**, where the subscripts *x* and *Std.* denote sample and standard, respectively, $Grad$ equals the slope of the plot of the integrated fluorescence intensity *vs* absorbance at the λ_{Ex} and η is the refractive index of the solvent.

$$\Phi_{F,x} = \Phi_{F,Std.} \times \left(\frac{Grad_x}{Grad_{Std.}}\right) \times \left(\frac{{\eta_x}^2}{{\eta_{Std.}}^2}\right) \qquad \textbf{Equation S2}$$

The fluorescence quantum yields of the standard and the refractive indexes of the solvents used in the calculations were obtained from the literature and are outlined below:

Cresyl violet in MeOH after excitation at 546 or 578 nm ($\Phi_F = 0.55$) [2].

Solvents: MeOH (η = 1.3288), ACN (η = 1.3442), DCM (η = 1.4242) [3].

4.4. Photostability of COUPY dyes.

The photostability of the compounds was assessed by monitoring the fluorescence intensity at the emission maximum wavelength (λ_{Em}) upon excitation at 560 nm of a 20 µM aqueous solution of the corresponding dye (40 µM in the case of coumarin **3**) after light irradiation using a custom-built irradiation setup from Microbeam, which includes a cuvette, a thermostated cuvette holder, and a high-power LED of wide range (470-750 nm range, centred at 530 nm; 150 mW/cm^2) light equipped with a bandpass filter that affords yellow light with a maximum emission wavelength centred around 560±40 nm (40 mW/cm^2). The aqueous solutions of the various coumarin derivatives were irradiated for 10 min using pulses of 30 sec. An emission spectrum was recorded after each irradiation pulse. The ratio of the fluorescence intensity at the λ_{Em} at each timepoint by the fluorescence intensity at the λ_{Em} before irradiation was plotted

against the light fluence (J/cm^2) using GraphPad Prism version 9.00 for Windows (GraphPad Software Inc., La Jolla, CA, USA).

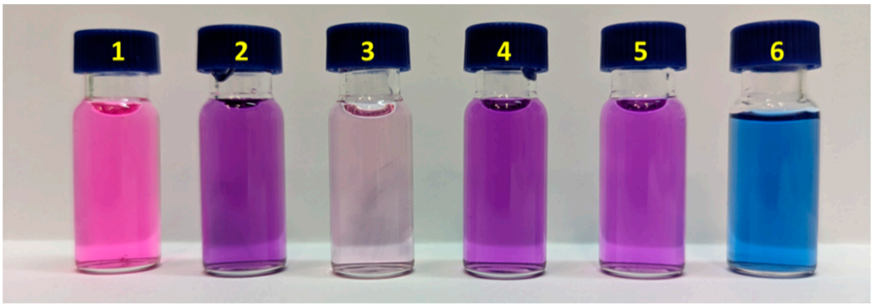

Figure S16. Photographic images of coumarin derivatives **1-6** (20 μM) in H_2O.

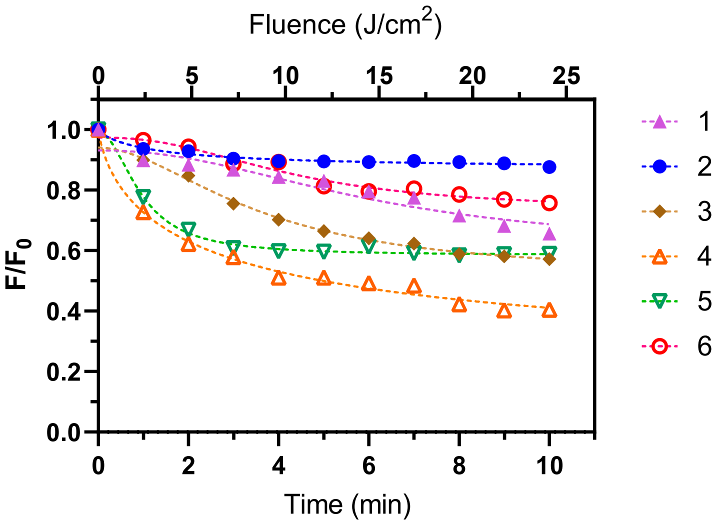

Figure S17. Fluorescence bleaching of aqueous solutions of COUPY **1-6** irradiated with yellow light (560±40 nm, 40 mW/cm^2).

5. Confocal microscopy studies

5.1. Cell Culture and treatments.

HeLa cells (American Type Culture Collection; Manassas, VA, USA) were maintained in DMEM (Dulbecco's Modified Eagle Medium) containing high glucose (4.5 g/L) and supplemented with 10% foetal bovine serum (FBS) and 50 U/mL penicillin−streptomycin. For cellular uptake experiments and subsequent observation under a microscope, cells were seeded on glass-bottom dishes (P35G-1.5-14-C, Mattek). Twenty-four hours after seeding, the cells were incubated for 30 minutes at 37 °C with a 2 μM dilution of the respective COUPY dye in non-supplemented DMEM without phenol red, prepared from a 10 mM stock solution in DMSO. Then, the cells were washed three times with DPBS (Dulbecco's phosphate buffered saline, pH 7.0−7.3) to remove the excess fluorophores and kept in low glucose DMEM without phenol red for fluorescence imaging.

For colocalization experiments of COUPY dyes with MitoTracker Green FM (MTG) and Hoechst 33342, HeLa cells were incubated for 30 min at 37 ºC with the corresponding COUPY dye (2 μM), MTG (1 μM) and/or Hoechst 33342 (1 μg/mL) in non-supplemented DMEM without phenol red, 24 hours after cell seeding. Then, the cells were washed three times with DPBS and kept in low glucose DMEM without phenol red for fluorescence imaging.

5.2. Fluorescence Imaging.

All microscopy observations were performed using a Zeiss LSM 880 confocal microscope equipped with a 405 nm laser diode, an argon-ion laser, a 561 nm laser, and a 633 nm laser. The microscope was also equipped with a Heating Insert P S (Pecon) and a 5% CO2 providing system. Cells were observed using a 63×1.4 oil-immersion objective. COUPY dyes were excited using the 561 nm laser and detected from 575 to 650 nm, or the 633 nm laser and detected from 645 to 750 nm. In colocalization studies, MitoTracker Green FM was observed using the 488 nm laser line of the argon-ion laser and detected from 500 to 550 nm, whereas Hoechst was excited with the 405 nm laser diode and detected from 410 to 480 nm. All observations were performed using laser powers in the range of μW.

5.3. Image analysis

Image processing and analysis was performed using Fiji [4]. COUPY dyes images were processed by filtering with a median filter of radius 1 and background subtracted with a rolling ball of 10.

Intensity measurements of the mitochondria stained by COUPY dyes **4** and **5** were performed on the maximum intensity projections (MIP) of the image stacks. After projecting and processing as described above, mitochondria were segmented using a random forest classifier trained with the Labkit plugin [5].

In the colocalization studies of the COUPY dyes with MTG, all stainings were processed identically as described above. Otsu intensity threshold [6] was then checked in each stack to finally apply it to the JaCoP plugin [7] to analyse the Pearson's correlation and Manders' colocalization coefficients. In all cases, Manders M1 and M2 coefficients corresponded to compound colocalizing over MTG and MTG over compound, respectively.

5.4. Additional confocal microscopy results:

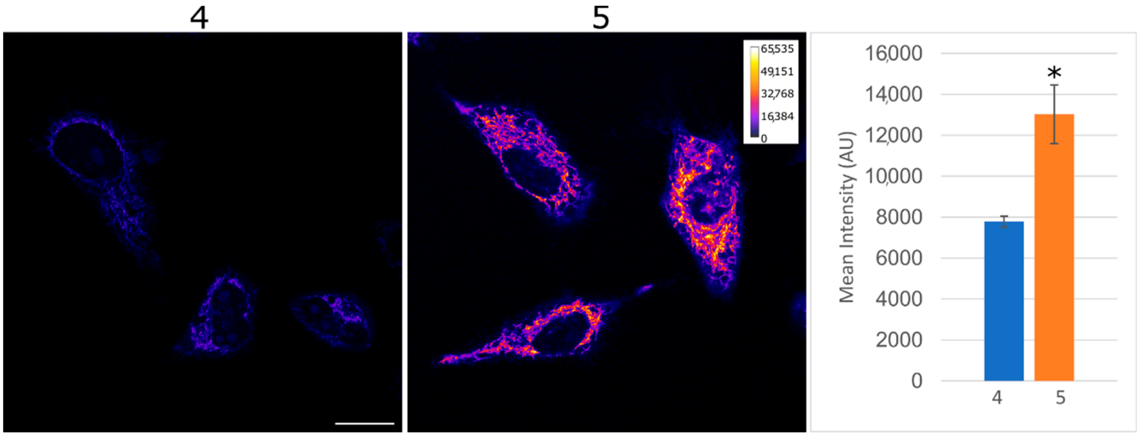

Figure S18. Cellular uptake of COUPY dyes **4** and **5** in HeLa cells. Single confocal planes of HeLa cells treated for 30 min at 37 ºC with compound **4** (left) or **5** (middle) at a final concentration of 2 μM, after excitation at 561 nm. Fluorescent signal is shown in Fire LUT; see calibration bar on the right top of the middle image. Scale bar: 20 μm; both two images are at the same scale. Right: comparison of the fluorescence signal of dyes **4** and **5** using the same excitation and detection settings. Statistical significance obtained using the t-test (*: p< 0.01). Data represented as mean±SD (n=28 cells).

Table S1. Colocalization coefficients between COUPY dyes **4-6** and Mitotracker Green FM. PCC is Pearson's correlation coefficient; M1 and M2 are Manders' coefficients: compound over organelle staining and organelle staining over compound, respectively. n ≥ 34 cells.

Compound	PCC	M1	M2	n-Cells
**4**	0.80	0.73	0.77	43
**5**	0.81	0.77	0.75	34
**6**	0.77	0.69	0.77	45

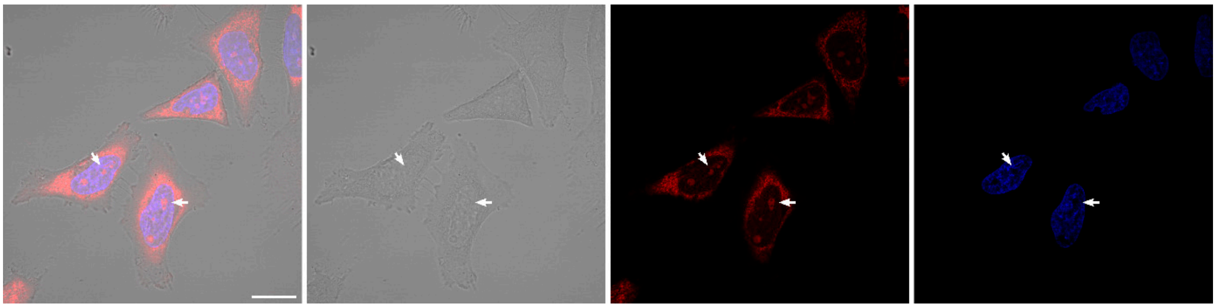

Figure S19. Co-localization studies of COUPY dye **4** with Hoechst 33342. Single confocal plane of HeLa cells incubated with **4** (2 µM, λ_{Ex}=561 nm, red) and Hoechst 33342 (1 µg/ml, λ_{Ex}=405 nm, blue) both for 30 min at 37 ºC. From left to right: merged images, brightfield, COUPY dye **4** and Hoechst 33342 single images, respectively. White arrows point out some nucleoli. Scale bar: 20 µm.

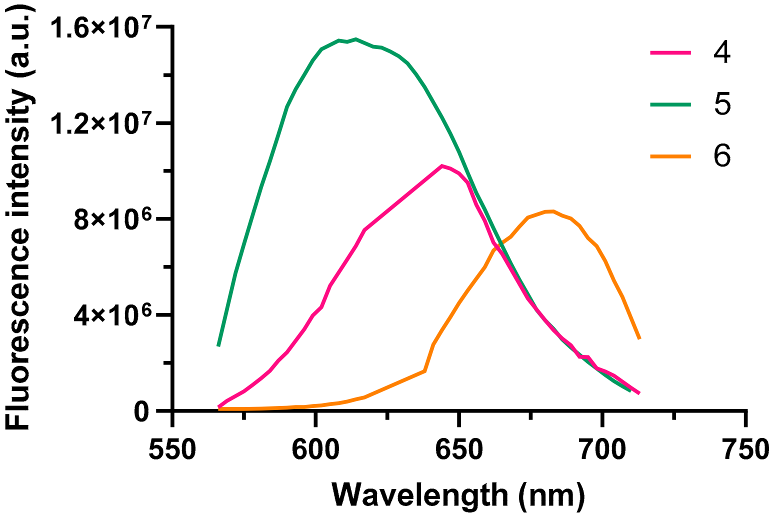

Figure S20. Emission spectra of COUPY dyes **4-6** upon excitation at 561 nm recorded in live HeLa cells.

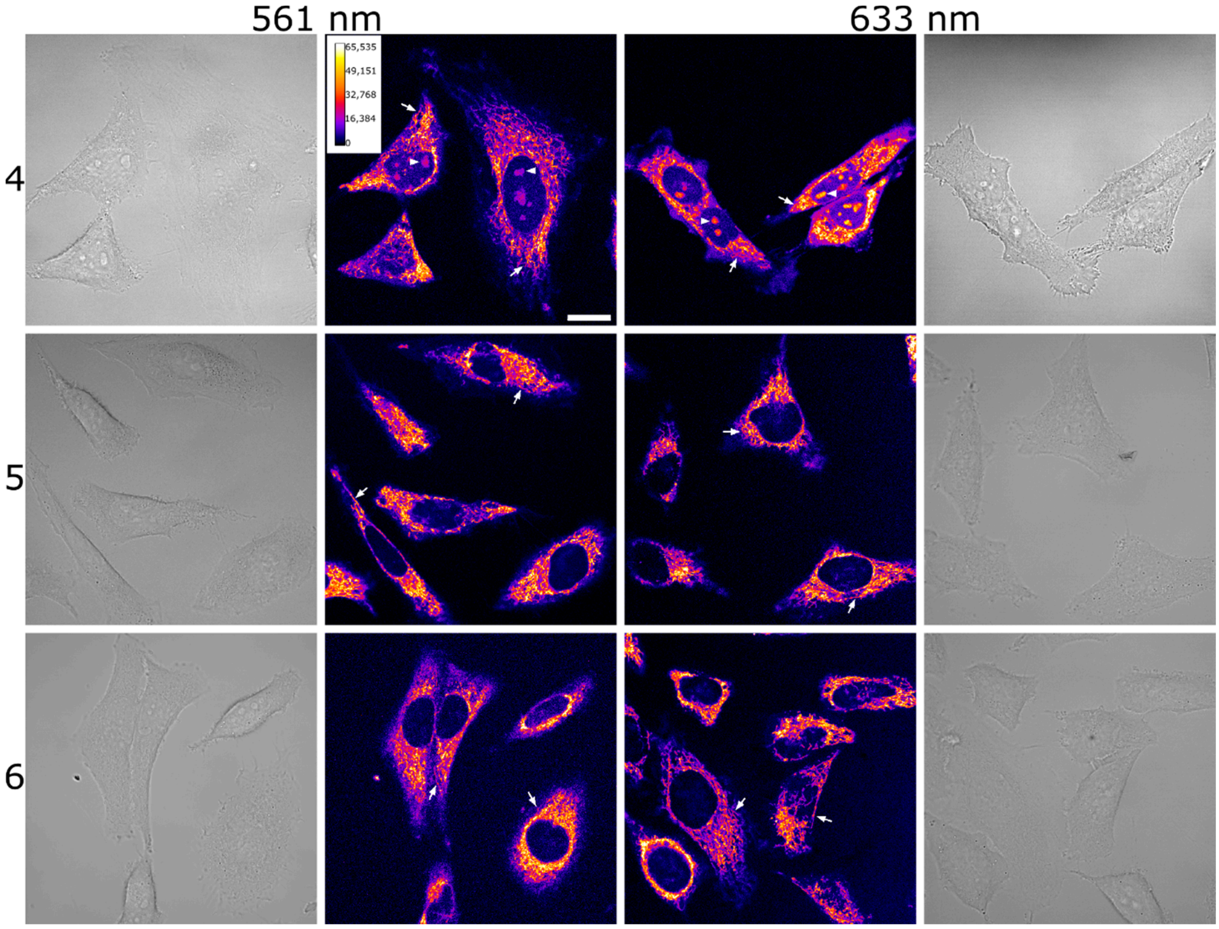

Figure S21. Cellular uptake of COUPY dyes **4**, **5** and **6**. Single confocal planes of HeLa cells treated for 30 min at 37 ºC with compound **4** (top), **5** (middle) or **6** (bottom) at a final concentration of 2 µM, after excitation at 561 nm (left two panels) or 633 nm (right two panels). Left and right columns show brightfield images. Fluorescent images in central two columns show compound signal in Fire LUT; the calibration bar is shown on the top left corner of compound **4** image. White arrows point out mitochondria, white arrowheads in top row point out nucleoli. Scale bar: 20 µm; all images are at the same scale. All fluorescent images are the same as those shown in Figure 3, but include the corresponding brightfield images.

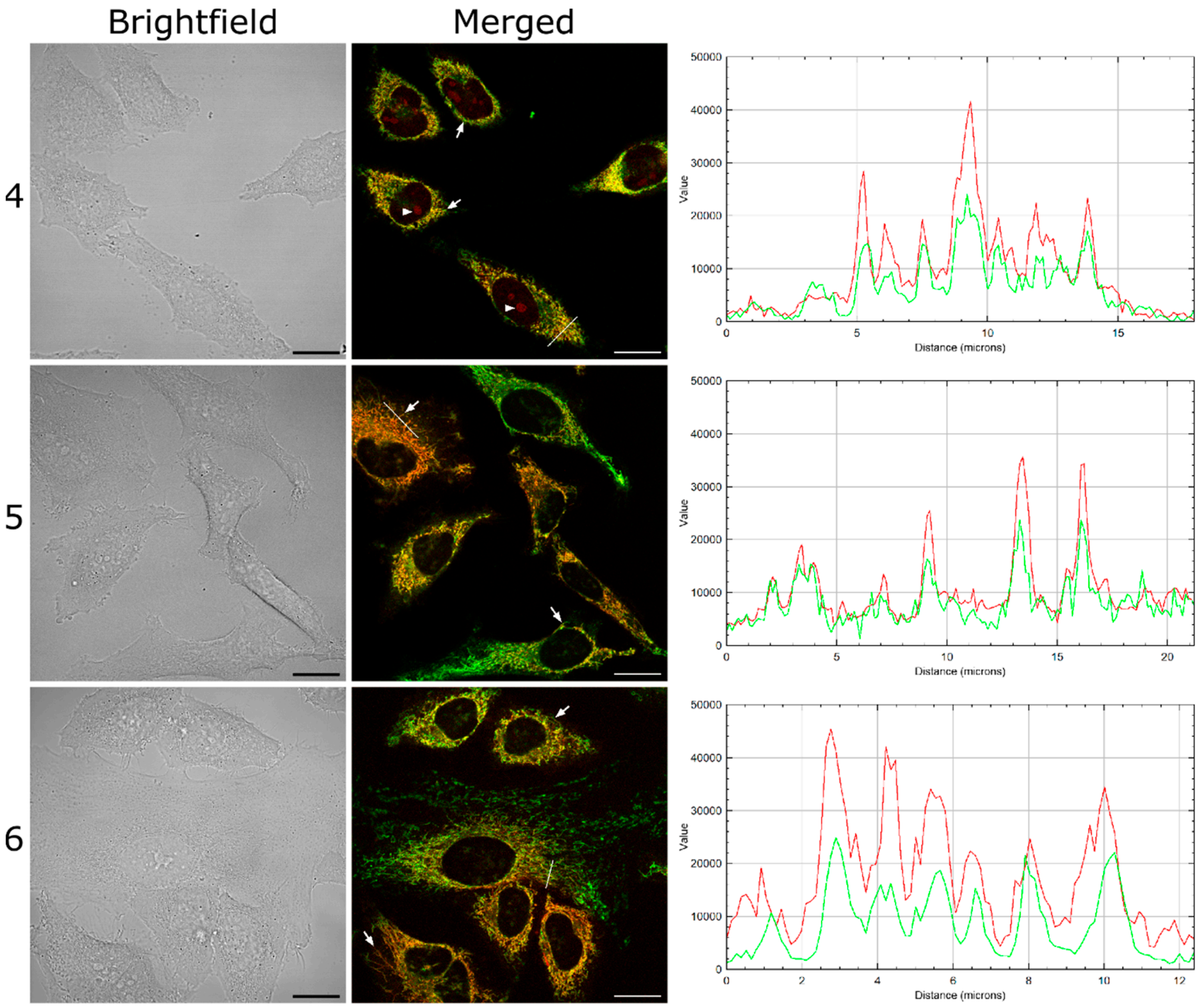

Figure S22. Co-localization studies of COUPY dyes **4** (top), **5** (middle) and **6** (bottom) with MitoTracker Green FM (MTG). Single confocal planes of HeLa cells incubated with the corresponding coumarin dye (2 µM, λ_{Ex}=561 nm, λ_{Em}=575-650 nm, red) and MTG (1 µM, λ_{Ex}=488 nm, λ_{Em}=500-550 nm, green) both for 30 min at 37 ºC. Left: brightfield images; center: merged images; right: intensity profile graphs obtained from white lines drawn on merged images. White arrows point out some colocalizing mitochondria, white arrowheads in top row point out nucleoli. Scale bar: 20 µm; all images are at the same scale. All fluorescent images are the same as those shown in Figure 4, but include the corresponding brightfield images.

6. References

1. Resch-Genger, U.; Rurack, K. Determination of the Photoluminescence Quantum Yield of Dilute Dye Solutions (IUPAC Technical Report). *Pure Appl. Chem.* **2013**, *85*, 2005–2013.

2. Brouwer, A.M. Standards for Photoluminescence Quantum Yield Measurements in Solution (IUPAC Technical Report). *Pure Appl. Chem.* **2011**, *83*, 2213–2228.

3. Saunders, J.E.; Sanders, C.; Chen, H.; Loock, H.-P. Refractive Indices of Common Solvents and Solutions at 1550 nm. *Appl. Opt.* **2016**, *55*, 947–953.

4. Schindelin, J.; Arganda-Carreras, I.; Frise, E.; Kaynig, V.; Longair, M.; Pietzsch, T.; Preibisch, S.; Rueden, C.; Saalfeld, S.; Schmid, B.; Tinevez, J.-Y.; White, D.J.; Hartenstein, V.; Eliceiri, K.; Tomancak, P.; Cardona, A. Fiji: An Open-Source Platform for Biological-Image Analysis. *Nat. Methods* **2012**, *9*, 676–682.

5. Arzt, M.; Deschamps, J.; Schmied, C.; Pietzsch, T.; Schmidt, D.; Tomancak, P.; Haase, R.; Jug, F. LABKIT: Labeling and Segmentation Toolkit for Big Image Data. *Front. Comput. Sci.* **2022**, *4*, 1–12.

6. Otsu, N. A Threshold Selection Method from Gray-Level Histograms. *IEEE Trans. Syst. Man. Cybern.* **1979**, *9*, 62–66.

7. Bolte, S.; Cordelières, F.P. A Guided Tour into Subcellular Colocalization Analysis in Light Microscopy. *J. Microsc.* **2006**, *224*, 213–232.
